# Oncolytic adenoviruses expressing checkpoint inhibitors for cancer therapy

**DOI:** 10.1038/s41392-023-01683-2

**Published:** 2023-11-29

**Authors:** Daoyuan Xie, Yaomei Tian, Die Hu, Yuanda Wang, Yuling Yang, Bailing Zhou, Rui Zhang, Zhixiang Ren, Mohan Liu, Jie Xu, Chunyan Dong, Binyan Zhao, Li Yang

**Affiliations:** 1 Department of Biotherapy, Cancer Center and State Key Laboratory of Biotherapy, West China Hospital, Sichuan University, Chengdu, 610041 China; 2 College of Bioengineering, Sichuan University of Science & Engineering, Zigong, 643000 China; 3 Frontiers Medical Center, Tianfu Jincheng Laboratory, Chengdu, 610212 China

**Keywords:** Drug development, Tumour immunology

## Abstract

Despite the remarkable success of immune checkpoint inhibitors (ICIs), primary resistance to ICIs causes only subsets of patients to achieve durable responses due to the complex tumor microenvironment (TME). Oncolytic viruses (OVs) can overcome the immunosuppressive TME and promote systemic antitumor immunity in hosts. Engineered OVs armed with ICIs would likely have improved effectiveness as a cancer therapy. According to the diverse immune cell landscapes among different types of tumors, we rationally and precisely generated three recombinant oncolytic adenoviruses (OAds): OAd-SIRPα-Fc, OAd-Siglec10-Fc and OAd-TIGIT-Fc. These viruses were designed to locally deliver SIRPα-Fc, Siglec10-Fc or TIGIT-Fc fusion proteins recognizing CD47, CD24 or CD155, respectively, in the TME to achieve enhanced antitumor effects. Our results suggested that OAd-SIRPα-Fc and OAd-Siglec10-Fc both showed outstanding efficacy in tumor suppression of macrophage-dominated tumors, while OAd-TIGIT-Fc showed the best antitumor immunity in CD8^+^ T-cell-dominated tumors. Importantly, the recombinant OAds activated an inflammatory immune response and generated long-term antitumor memory. In addition, the combination of OAd-Siglec10-Fc with anti-PD-1 significantly enhanced the antitumor effect in a 4T1 tumor model by remodeling the TME. In summary, rationally designed OAds expressing ICIs tailored to the immune cell landscape in the TME can precisely achieve tumor-specific immunotherapy of cancer.

## Introduction

Immunotherapy has played an increasingly important role in tumor therapy over the past several years. Cancer immunotherapy targeting T-cell checkpoint axes using ICIs has demonstrated the power of unleashing antitumor cytotoxic T-cell activity and has led to remarkable success in the clinic.^1^ In particular, antibodies targeting CTLA4 and the PD1/PDL1 axis have been approved for use in several cancer types.^2^ However, an increasing number of cases have indicated that only a subset of patients benefits from ICI treatment owing to deficiencies in antigen presentation or a low number of CD8^+^ effector T cells in the immunosuppressive TME.^3^ Emerging evidence suggests that the composition and spatial organization of tumor-infiltrating immune cells across cancer types serves as a major tumor-intrinsic factor that affects the efficacy of ICI therapy.^4,5^

Tumor-infiltrating immune cells play an important role in the regulation of antitumor immunity. However, there are significant differences in the composition and functional state of tumor-infiltrating immune cells across tumor types.^6^ Early lung adenocarcinoma shows high accumulation of lymphocytes and clonal expansion of CD8^+^ PD-1^+^ T cells at the tumor site, which likely contribute to the success of anti-PD-1 checkpoint blockade in these patients.^6,7^ Pancreatic ductal adenocarcinoma shows dominant infiltration by macrophages, especially the M2 type.^8,9^ Colorectal cancer and brain cancers have relatively high fractions of monocytes/macrophages compared to those of T cells and natural killer (NK) cells.^10^ These factors may confer poor clinical responses to ICI immunotherapy in patients. Therefore, based on the detection of the composition of infiltrated immune cells in the TME, targeting the dominant infiltrated immune cell population is a rational strategy to precisely suppress tumor growth rather than simply depending on blockade of CTLA4 or the PD1/PDL1 axis. For example, blockade of CD47-SIRPα or CD24-Siglec10 results in a macrophage-dependent reduction in tumor growth.^11,12^ Furthermore, developing additional therapies is essential to overcoming the limitations of ICI therapy, such as issues related to activating and recruiting immune cells in the TME.

OVs preferentially target and selectively replicate in the TME and can modify the immunosuppressive TME of multiple solid tumors.^13,14^ OVs possess the potential to induce immunogenic cell death, cause neoantigen release and presentation, and remodel the immunosuppressive TME with the assistance of concurrently expressed damage- or pathogen-associated molecular patterns, making these viruses promising candidates for in situ vaccination against tumors.^13,15^ And also, a series of methods have been developed to achieve systemic administration of oncolytic viruses.^16,17^ In addition, OVs can deliver immunomodulators into the tumor bed to further enhance antitumor immune responses with minimal systemic toxicity, for example, by delivering an anti-PD-1 antibody^18,19^ or a CTLA4-specific ScFv.^20^ Moreover, the PD-L1 upregulation that occurs in the TME after virus administration enhances the response to ICI treatment.^21^ However, in a triple-negative breast cancer model, the upregulation of PD-L1 expression on cancer cells resulted in immune escape.^22^ Therefore, the combination of an OV and anti-PD-1 may be able to overcome immunosuppression in the TME.

In this study, based on the landscape of infiltrated immune cells in the TME, we generated engineered OAds carrying transgenes encoding the extracellular domain of SIRPα or Siglec10 on the Fc scaffold to target dominant macrophages or encoding that of TIGIT to target dominant T cells (termed OAd-SIRPα-Fc, OAd-Siglec10-Fc, and OAd-TIGIT-Fc, respectively). In our evaluation, OAd-SIRPα-Fc and OAd-Siglec10-Fc preferentially suppressed tumors strongly dominated by macrophages, while OAd-TIGIT-Fc preferentially targeted tumors with a high T-cell fraction. In a triple-negative breast cancer tumor model, the antitumor effect of OAd-Siglec10-Fc was enhanced by combining OAd-Siglec10-Fc with anti-PD-1 to remodel the TME. In summary, these attributes hold promise for the rational clinical development of OVs to precisely treat cancers.

## Results

### Immune cell typing across human and mouse tumors

Sixty to seventy percent of patients do not respond to anti-PD-1 therapy due to the complexity of the TME.^23^ The next wave of co-inhibitory targets, including CD24, CD47, CD155, LAG3, CD276, CD39, CD73, adenosine A2A receptor, etc. is being explored in clinical development. Targeting immune checkpoints in macrophages could restore the phagocytic activity of macrophages and prevent tumor relapse and progression.

**Fig. 1:**
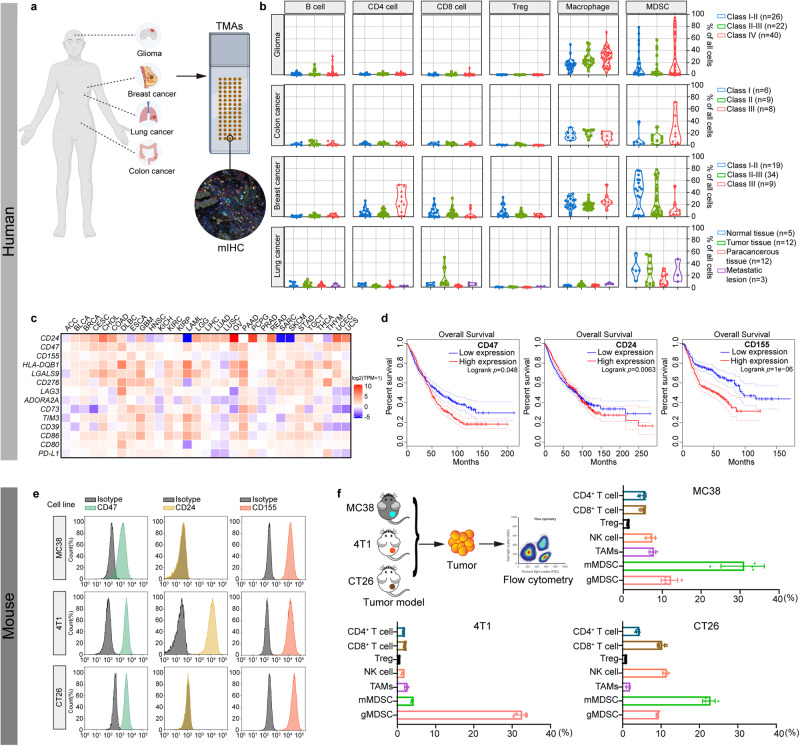
Immune cell typing across human and mouse TMEs. **a**, **b** The immune landscape was identified by mIHC staining of human glioma, colon cancer, breast cancer and lung cancer tumor microarrays. **c**, **d** Heatmap of immune checkpoint molecules in tumors to matched normal expression ratios (log2 (TPM + 1)) (**c**) and OS of patients with tumors (**d**). **e** The expression of CD24, CD47 and CD155 in MC38, 4T1, and CT26 tumor cells was detected by flow cytometry. **f** C57BL/6J mice were subcutaneously inoculated with 1 × 10^6^ MC38 cells. BALB/c mice were subcutaneously inoculated with 1 × 10^6^ 4T1 cells or 1.5 × 10^6^ CT26 cells. When tumor sizes reached ~100 mm^3^, single-cell suspensions were prepared from the mouse tumors prior to analyzing the composition of immune cells by flow cytometry. Data are represented as mean ± SD

Therefore, ICI therapy should be based on the main population of infiltrating mononuclear cells, such as T cells or macrophages. We first explored the immune landscapes of four types of tumors, including glioma, colon cancer, breast cancer and lung cancer. Tumor microarrays of glioma (*n* = 88), colon cancer (*n* = 23), breast cancer (*n* = 62) and lung cancer (*n* = 24) samples were used to identify B cells (anti-CD20), CD4^+^ T cells (anti-CD4), CD8^+^ T cells (anti-CD8), regulatory T cells (Tregs, anti-Foxp3), macrophages (anti-CD68) and myeloid-derived suppressor cells (MDSCs, anti-Arginase-1) by multiplex immunohistochemistry (mIHC) (Fig. 1a and Supplementary Fig. 1a–d). MDSCs were the most abundant constituent of all four types of tumors (Fig. 1b). Glioma, colon cancer and breast cancer had a higher proportion of macrophages than CD8^+^ T cells, but a high proportion of CD8^+^ T cells was observed in lung cancer (Fig. 1b). We further examined the expression of immune checkpoints (CD24, CD47, CD155, HLA-DQB1, LGALS9, CD276, LAG3, ADORA2A, CD73, TIM3, CD39, CD80, CD86, and PD-L1) in various tumors. RNA-seq data from TCGA (http://www.cbioportal.org/) revealed high expression of CD24, CD47, and CD155 in most cancers (Fig. 1c and Supplementary Table 1). The mRNA expression of CD24, CD47 and CD155 was further verified in colon adenocarcinoma (COAD), liver hepatocellular carcinoma (LIHC), lung adenocarcinoma (LUAD) and stomach adenocarcinoma (STAD) patient samples via cDNA microarray. The results showed that the mRNA expression of CD24, CD47, and CD155 was presented in these four cancer types, which was consistent with TCGA results (Supplementary Fig. 2a). The correlation analysis between the immune checkpoints and the immune cells was further studied. Increased expression of Siglec10, SIRPα, and TIGIT was significantly associated with the infiltration levels of neutrophils and dendritic cells (DCs) in almost all tumor types except THYM. In addition, the expression of Siglec10 and SIRPα had strong associations with macrophages while TIGIT had weak associations with macrophage among almost all tumor types. In addition, TIGIT had high relativity with CD8^+^ T cells (Supplementary Fig. 2b). High expression of CD24, CD47, and CD155 was associated with short overall survival (OS) in cancers (Fig. 1d). Based on the composition and phenotypic states of intratumoral immune cells in the different tumor types and even the expression of ligands, CD47 and CD24 were rationally selected as targets to restore macrophage-mediated phagocytosis in macrophage-dominated tumors, while CD155 was used as a target to prevent T-cell exhaustion in CD8^+^ T-cell-dominated tumors.

The murine cancer cell lines MC38, 4T1, and CT26 were chosen to investigate targeted therapies. MC38, 4T1, and CT26 cells all showed high expression of CD47 and CD155, but only 4T1 cells expressed CD24 (Fig. 1e). Furthermore, the landscapes of infiltrated immune cell types in the three tumors were different, with a high abundance of monocytic MDSC (mMDSC) in MC38 and CT26 tumors and a high abundance of granulocytic MDSC (gMDSC) in 4T1 tumors. In addition, tumor-associated macrophages (TAMs) accounted for a large proportion of immune cells in 4T1 and MC38 tumors, but CD8^+^ T cells and NK cells predominated in CT26 tumors (Fig. 1f). Although highly abundance of MDSCs (including gMDSC and mMDSC) were found in TME, but macrophage-targeting strategies show better therapeutic efficacy than MDSCs.^24^ Therefore, using SIRPα-Fc to block CD47 or Siglec10-Fc to block CD24 in macrophage-dominated MC38 and 4T1 tumors or TIGIT-Fc to block CD155 in CD8^+^ T-cell-dominated CT26 tumors may be a rational strategy for cancer immunotherapy.

### Generation and characterization of OAd-SIRPα-Fc, OAd-Siglec10-Fc, and OAd-TIGIT-Fc

**Fig. 2:**
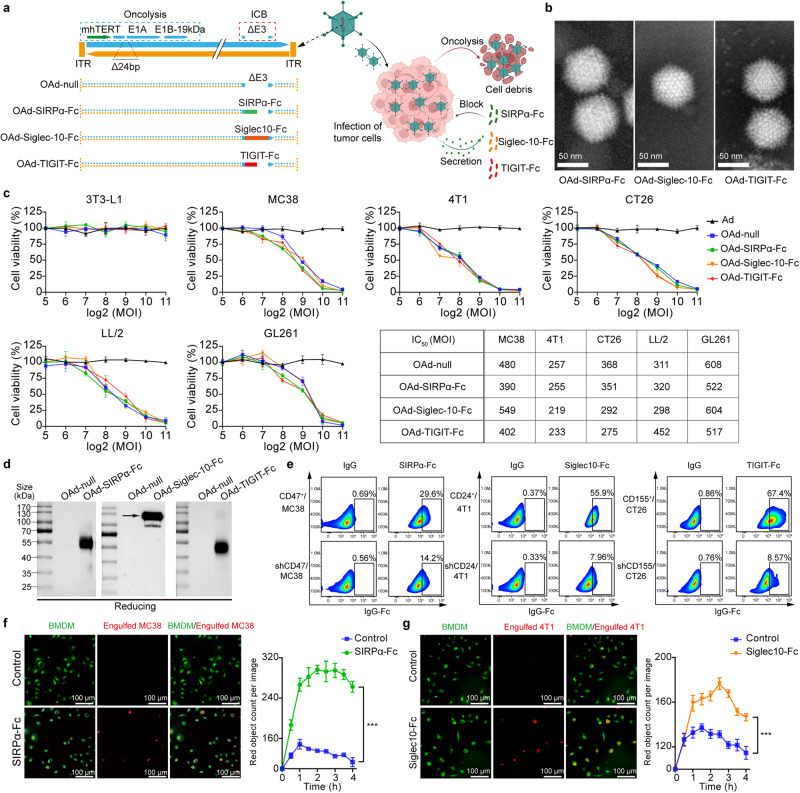
Generation and characterization of OAd-SIRPα-Fc, OAd-Siglec10-Fc, and OAd-TIGIT-Fc. **a** Schematic representation of OAd structures. **b** Transmission electron microscopy view of OAd-SIRPα-Fc, OAd-Siglec10-Fc, and OAd-TIGIT-Fc (scale bar: 50 nm). **c** The oncolytic potency of OAd-SIRPα-Fc, OAd-Siglec10-Fc, and OAd-TIGIT-Fc was evaluated against MC38, 4T1, CT26, LL/2, and GL261 tumor cells. Nontumor 3T3-L1 cells were used as a negative control. Ad is a replication-deficient adenovirus without the mhTERT promoter and E1A/E1B genes. **d** The expression and secretion of SIRPα-Fc, Siglec10-Fc, and TIGIT-Fc into the supernatant from the indicated OAd-infected MC38 tumor cells were detected by western blotting under reducing conditions. **e** Wild-type cells or ligand-knockdown cells were incubated with purified SIRPα-Fc, Siglec10-Fc and TIGIT-Fc from corresponding virus-infected tumor cells and then stained with anti-IgG Fc for flow cytometric detection. IgG acted as a negative control. **f**, **g** HEK293 cells were infected with OAd-SIRPα-Fc or OAd-Siglec10-Fc for 72 h, and then the supernatant was collected. pHrodo (red)-labeled MC38 (**f**) or 4T1 cells (**g**) were incubated with supernatants containing SIRPα-Fc or Siglec10-Fc for 1 h and then cocultured with CDFA-SE-labeled M1-BMDMs. Colocalization of the two cell types demonstrated phagocytosis. The supernatant from OAd-null-infected HEK293 cells was used as a control. (***p < 0.001)

Tumor selectivity was conferred to replication-competent OAds by inserting a modified hTERT (mhTERT) promoter to drive the expression of the E1 gene in which a 24-bp sequence in the E1A region and an E1B55-kD viral protein in the E1B region were deleted (Fig. 2a).^25,26^ The mhTERT promoter produced significantly higher luciferase gene activity than the wild-type hTERT promoter (wt-hTERT) in mouse tumor cells (GL261, MC38, LL/2, 4T1, and CT26) but a low level of luciferase gene activity in normal mouse 3T3-L1 cells (Supplementary Fig. 3).

OAd-SIRPα-Fc, OAd-Siglec10-Fc and OAd-TIGIT-Fc were engineered by introducing the murine soluble SIRPα, Siglec10 or TIGIT extracellular domains, respectively, fused with IgG1 Fc by the CMV promoter into the ∆E3 region of OAd-null, which had oncolytic and immune checkpoint blockade functions (Fig. 2a). The hexagonal structures and their fiber dots on the surface of purified OAds were clearly visible by transmission electron microscopy (Fig. 2b). These engineered OAds were able to selectively kill multiple mouse tumor cell lines, demonstrating that the insertion of transgene cassettes did not interfere with the infection or replication of OAds in vitro (Fig. 2c). Immunoblot analysis showed that tumor cells infected with OAd-SIRPα-Fc, OAd-Siglec10-Fc or OAd-TIGIT-Fc efficiently secreted the corresponding fusion protein as a dimer into the supernatant in vitro (Fig. 2d and Supplementary Fig. 4). To determine the binding affinities of secreted SIRPα-Fc, Siglec10-Fc and TIGIT-Fc for their ligands, we purified these proteins from the supernatants of corresponding virus-infected tumor cells and constructed CD47-knockdown MC38 cells, CD24-knockdown 4T1 cells and CD155-knockdown CT26 cells using shRNA (Supplementary Fig. 5). Purified SIRPα-Fc was capable of binding to CD47^+^ MC38 cells but not to CD47-knockdown cells (Fig. 2e). Similarly, the results of the Siglec10-Fc and TIGIT-Fc binding assays demonstrated specific affinity for the corresponding ligand (Fig. 2e). Furthermore, the specific binding of purified SIRPα-Fc and Siglec10-Fc to CD47 or CD24, respectively, enhanced macrophage‐mediated tumor cell phagocytosis in vitro (Fig. 2f, g). Taken together, these data demonstrate that the armed OAd-SIRPα-Fc, OAd-Siglec10-Fc and OAd-TIGIT-Fc can selectively lyse tumor cells and secrete high levels of functional fusion proteins.

### Precise antitumor activities against primary tumors

**Fig. 3:**
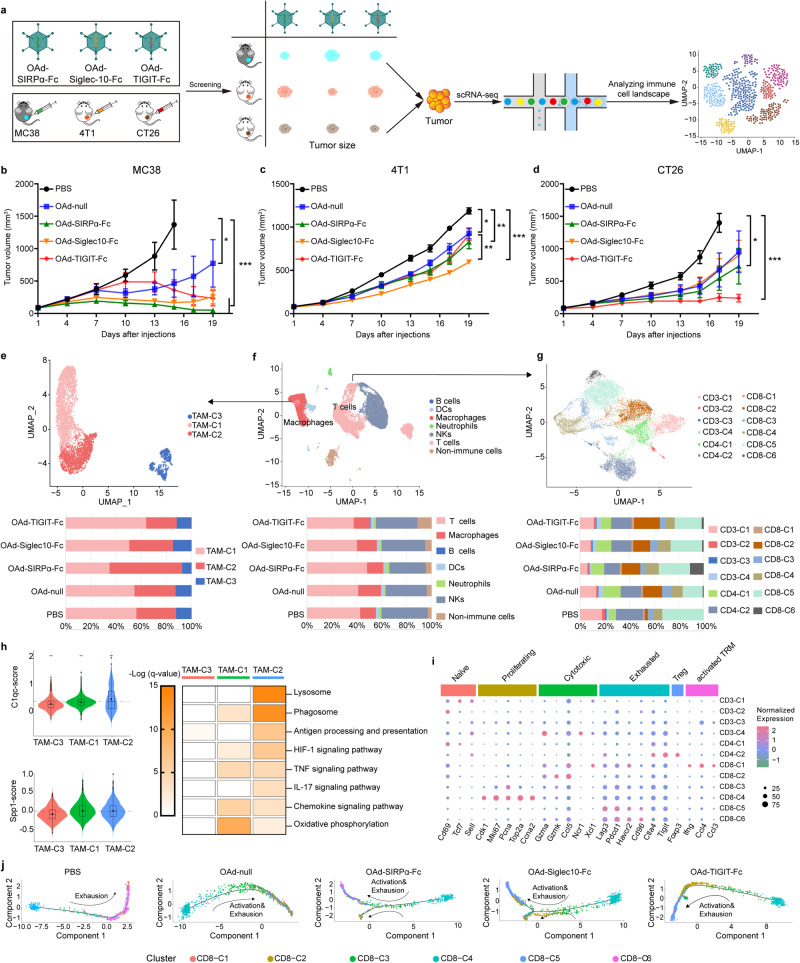
Assessment of the functional states of tumor-infiltrating TAMs and T cells in the MC38 model. **a** Schematic flow diagram of the precision treatment strategy with different OAds for different tumor types. **b**–**d** Tumor-bearing mice were intratumorally injected with 50 μL of OAds (1 × 10^8^ pfu per tumor) for the MC38 (**b**) and CT26 models (**d**) or with OAds (3 × 10^8^ pfu per tumor) for the 4T1 model (**c**) on days 1, 4, 7, 10 and 13. The treatment regimens for PBS, Ad and OAd-null in the corresponding models were consistent with those for the armed OAds. Tumor volume was monitored. **e**–**g** MC38 tumor-bearing mice were treated with PBS, OAd-null, OAd-SIRPα-Fc, OAd-Siglec10-Fc, or OAd-TIGIT-Fc. Two days after the third OAd dose, tumor issues were profiled by scRNA-seq. UMAP plot of all single cells in the MC38 model and histogram indicating the proportions of cell clusters in tumor tissues (**f**). UMAP plots of identified macrophages and frequencies of macrophage subsets from the scRNA-seq analysis (**e**). UMAP plots of identified T cells and frequencies of T-cell subsets from the scRNA-seq analysis (**g**). **h** Violin plots showing comparisons of C1qc^+^ and Spp1^+^ score levels among macrophage subclusters. Heatmap of enriched KEGG pathways in TAM clusters. **i** Relative average expression of canonical marker genes across different T-cell clusters. **j** Developmental trajectory of CD8^+^ T cells inferred by Monocle 2. (*p < 0.05, **p < 0.01, ***p < 0.001)

Our results demonstrated that MC38 and 4T1 tumors were rich in macrophages, while CT26 tumors were rich in CD8^+^ T cells and NK cells. Moreover, OAd-SIRPα-Fc and OAd-Siglec10-Fc were used to target macrophages. OAd-TIGIT-Fc was employed to target CD8^+^ T cells and NK cells. To characterize the precise effects of OAds, we evaluated antitumor activity in three tumor models established with immunocompetent mice treated with an intratumoral injection of OAd-null, OAd-SIRPα-Fc, OAd-Siglec10-Fc, or OAd-TIGIT-Fc. PBS acted as a control (Fig. 3a).

OAd-null treatment markedly reduced tumor sizes compared with PBS treatment in the MC38 and 4T1 models (Fig. 3b, c). OAd-SIRPα-Fc treatment conferred better antitumor activity than OAd-Siglec10-Fc and OAd-TIGIT-Fc in the MC38 model (Fig. 3b), but OAd-Siglec10-Fc showed satisfactory tumor suppression in the 4T1 model (Fig. 3c). However, OAd-Siglec10-Fc and OAd-null had similar efficacies, which was likely caused by the lack of CD24 expression in the CT26 model (Fig. 3d). Compared with the other treatments, OAd-TIGIT-Fc showed the best antitumor activity in the CT26 model (Fig. 3d). These data demonstrated that SIRPα-Fc blocking of CD47 or Siglec10-Fc blocking of CD24 significantly suppressed the growth of tumors with a macrophage-dominated TME, while TIGIT-Fc blocking of CD155 showed promising antitumor activity in CD8^+^ T-cell- and NK cell-dominated tumors.

To better understand the mechanism associated with precise tumor regression achieved with the different OAd treatments, we performed comprehensive and unbiased scRNA-seq of MC38 and CT26 tumors to examine the cellular transcriptomic changes in the TME. Two days after the third injection, tumors were collected, and scRNA-seq analysis was performed on MC38 and CT26 tumors treated with PBS, OAd-null, OAd-SIRPα-Fc, OAd-Siglec10-Fc, or OAd-TIGIT-Fc. Following gene expression normalization for read depth and mitochondrial read count, we obtained high-quality expression data for 33758 cells from MC38 tumors and 30215 cells from CT26 tumors.

In MC38 tumors, after unbiased cell type classification using Seurat v4, 7 cell clusters were identified based on marker gene expression; these clusters included Non-immune cells, B cells, T cells, macrophages, neutrophils, and NK cells (Fig. 3f and Supplementary Fig. 6a). Compared to PBS-treated tumors, tumors treated with one of the four OAd treatments, especially OAd-SIRPα, had increased proportions of T cells (Fig. 3f). We calculated scores for macrophage, T-cell and NK cell clusters by using the *Seurat* function *AddModuleScore* to analyze functional states (Supplementary Table 2). OAd-SIRPα-Fc and OAd-Siglec10-Fc showed higher C1qc^+^ scores than OAd-null (Supplementary Fig. 7a). We did not find a significant difference in the C1qc^+^ score between the OAd-null and OAd-TIGIT groups. However, OAd-SIRPα-Fc showed a lower Spp1^+^ score than OAd-null. Furthermore, OAds treatments showed higher cytotoxicity scores for the T-cell and NK cell clusters than PBS treatment (Supplementary Fig. 7b, c). However, OAd-SIRPα-Fc showed a lower exhaustion score for the T-cell and NK cell clusters (Supplementary Fig. 7b, c). These data indicated improvements in antitumor activity following OAd-SIRPα-Fc treatment.

Based on the expression of canonical markers, we annotated macrophages into three subtypes (TAM-C1, TAM-C2 and TAM-C3) (Fig. 3e and Supplementary Fig. 6b). TAM-C2 was enriched in OAds treatments, especially OAd-SIRPα-Fc, compared with PBS treatment. TAM-C1 was markedly decreased in the OAd-SIRPα-Fc group compared with the other four groups (Fig. 3e). To better understand the roles of these populations, we further calculated C1qc and Spp1 gene signatures (Fig. 3h). TAM-C1 showed a high Spp1^+^ score, while TAM-C2 showed a high C1qc^+^ score. Interestingly, we noticed significant enrichment of gene expression signatures in the proinflammatory phenotype, such as antigen processing and presentation, HIF-1 signaling, TNF-α signaling, and IL17 signaling, in TAM-C2 compared to TAM-C1 (Fig. 3h). Importantly, our data show that the lysosomal and phagosomal pathways of TAM-C2 were more enriched than those of TAM-C1, which possessed the characteristics of M2 macrophages indicated by enrichment of gene expression signatures of oxidative phosphorylation.^27^

We further performed unsupervised clustering of T cells and obtained 12 clusters: CD3-C1 to CD3-C4, CD4-C1 to CD4-C2, and CD8-C1 to CD8-C6 (Fig. 3g and Supplementary Fig. 6c). The abundance of CD3-C1, CD4-C2 and CD8-C5 was significantly decreased in the OAd treatments compared to the PBS treatment. However, the relative percentage of CD8-C2 in the OAd treatment groups was increased compared with that in the PBS group. Interestingly, the abundance of CD8-C6 was increased with only OAd-SIRPα-Fc treatment compared with other treatments. Based on the expression of canonical markers, we identified naive CD3 cells (CD3-C1; *TCF7*^*+*^ and *SELL*^*+*^), Tregs (CD4-C2; *Foxp3*^*+*^, *TIGIT*^*+*^, and *CTLA4*^*+*^), and terminally exhausted T cells (CD8-C5; *PDCD1*^*+*^, *LAG3*^*+*^, *HAVCR2*^*+*^, and *CD96*^*+*^), indicating that the immune microenvironment in PBS-treated tumors was skewed toward a tolerogenic milieu but that the immunosuppressive TME was relieved in the OAd groups (Fig. 3i). Importantly, CD8-C2 showed high expression levels of *GZMA*, *GZMK* and *CCL5*, representing cytotoxic T cells with a high cytotoxicity T-cell gene signature score but a low exhausted T-cell gene signature score (Supplementary Fig. 7d, e). Similarly, CD8-C6 showed high expression levels of *PDCD1*, *LAG3*, *HAVCR2* and *CD96*, representing terminally exhausted T cells with a high cytotoxicity T-cell gene signature score but a low exhausted T-cell gene signature score. CD8-C5 and CD8-C6 exhibited an activation-coupled exhaustion program. CD8^+^ T cells in the CD8-C1 showed high expression levels of *IFNG*, *CCL4*, and *CCL3* (Fig. 3i), thus representing activated non-circulating tissue-resident memory T cells/effector memory T cells.^28^

CD8^+^ T cells in the CD8-C3 and CD8-C4 clusters showed high expression levels of some cell proliferation marker genes, such as MKi*67*, *PCNA*, *CDK1*, *Top2a*, and *CCNA2*, indicating that these clusters represented proliferating T cells.^29^

Finally, we constructed a developmental trajectory of CD8^+^ T cells that was associated with the cytotoxicity and exhaustion scores of CD8^+^ T-cell clusters (Fig. 3j). Along the trajectory, T cells exhibited an exhaustion status with almost no activation in the PBS group. T cells in the OAd-TIGIT-Fc group exhibited increasing cytotoxic activity, ultimately followed by exhaustion. However, T cells in the OAd-null, OAd-SIRPα-Fc and OAd-Siglec10-Fc groups exhibited gradually increasing cytotoxic activity, which was accompanied by gradually increasing exhaustion. Moreover, some of the T cells in the OAd-SIRPα-Fc group developed into a memory population (Fig. 3j). These data demonstrated that OAds, especially OAd-SIRPα-Fc, enhanced the activation of proinflammatory TAMs and cytotoxic CD8^+^ T cells and alleviated immunosuppression in the MC38 tumor model.

In the analysis of CT26 tumor scRNA-seq, we obtained similar results (Supplementary Results, Supplementary Fig. 8 and Supplementary Fig. 9). These data demonstrated that OAd-TIGIT-Fc treatment mainly enhanced the activation of cytotoxic CD8^+^ T cells, and alleviated immunosuppression in the CT26 tumor model.

### Enhanced antitumor activities and activated immune cells in primary tumors

Our results demonstrated that OAd-SIRPα-Fc, OAd-Siglec10-Fc, and OAd-TIGIT-Fc showed precise antitumor efficacy against MC38, 4T1, and CT26 tumors, respectively.

**Fig. 4:**
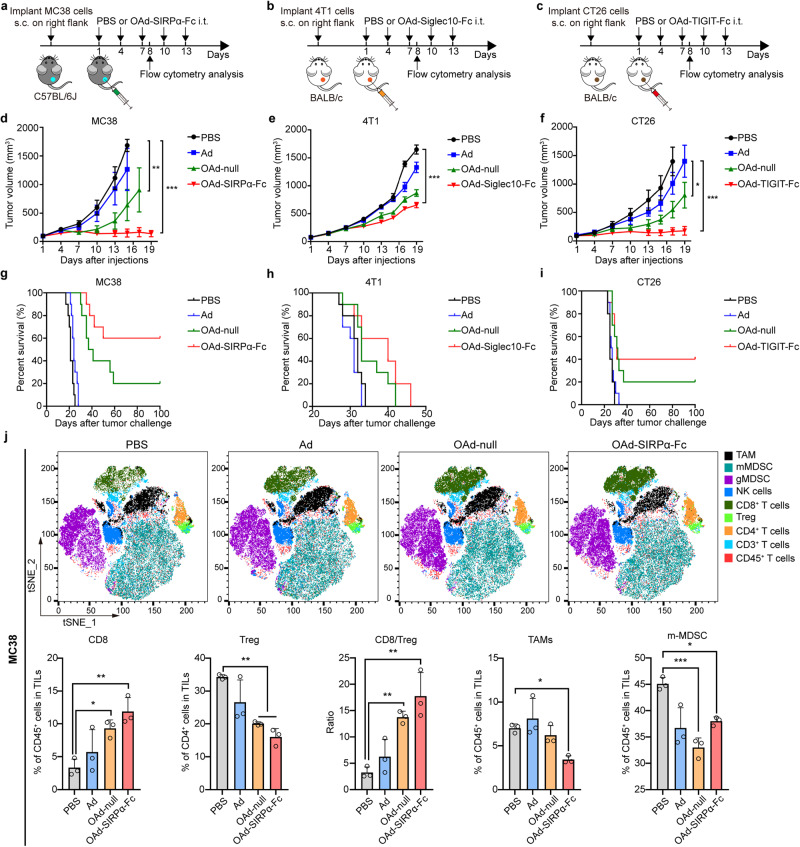
Enhanced antitumor activities against primary tumors. C57BL/6J mice were subcutaneously inoculated with 1 × 10^6^ MC38 cells. BALB/c mice were subcutaneously inoculated with 1 × 10^6^ 4T1 cells or 1.5 × 10^6^ CT26 cells. When tumor sizes reached ~100 mm^3^ (counted as day 1), the mice were intratumorally injected with 50 μL of OAd-SIRPα-Fc (1 × 10^8^ pfu per tumor) for the MC38 model (**a**), OAd-Siglec10-Fc (3 × 10^8^ pfu per tumor) for the 4T1 model (**b**), and OAd-TIGIT-Fc (1 × 10^8^ pfu per tumor) for the CT26 model (**c**) on days 1, 4, 7, 10, and 13. The treatment regimens for PBS, Ad or OAd-null in the corresponding models were consistent with those for the armed OAds. Tumor volume was monitored in mice bearing MC38 (**d**, *n* = 5), 4T1 (**e**, *n* = 7), or CT26 tumors (**f**, *n* = 5). Data are represented as mean ± SEM. Survival curves of mice bearing MC38 (**g**), 4T1 (**h**), and CT26 tumors (**i**) (n = 10). **j** Two days after the third injection, the treated tumors were collected and analyzed by flow cytometry to calculate the percentages of infiltrating CD8^+^ T cells, Tregs, TAMs and mMDSCs in MC38 tumors. *n* = 3 mice. Data are represented as mean ± SD. (*p < 0.05, **p < 0.01, ***p < 0.001)

Furthermore, their antitumor effects were assessed in these three different subcutaneous tumor models by analyzing tumor inhibition, survival time, and immunocyte infiltration in the TME. For the MC38, 4T1 and CT26 tumor models (Fig. 4a–c), intratumoral injection of OAd-null, OAd-SIRPα-Fc, OAd-Siglec10-Fc, or OAd-TIGIT-Fc in the corresponding model was significantly more potent than that of OAd-null regarding tumor growth inhibition (Fig. 4d–f) and survival prolongation (Fig. 4g–i), suggesting that SIRPα-Fc, Siglec10-Fc, and TIGIT-Fc were important contributors to the antitumor effect in addition to exerting direct oncolytic activity.

To further demonstrate the remodeling of the tumor immune microenvironment by OVs, we analyzed tumor-infiltrating lymphocytes by flow cytometry after the third intratumoral injection of various OAds (Fig. 4j and Supplementary Fig. 10). We quantified all the tumor-infiltrating immune cells as a percentage of total CD45^+^ cells in tumors (unless otherwise specified). The results showed that OAd-SIRPα-Fc injection enhanced the proportion of CD8^+^ T cells in MC38 tumor tissues more significantly than Ad or OAd-null injection (Fig. 4j). Similar increases in CD8^+^ T cell infiltration in 4T1 or CT26 tumor tissues were observed after OAd-Siglec10-Fc or OAd-TIGIT-Fc treatments (Supplementary Fig. 11). In addition, the percentage of Tregs in CD4^+^ T cells was significantly reduced after the injection of OAds compared with PBS treatment in all three models (Fig. 4j and Supplementary Fig. 11). Therefore, the injection of OAd-null, OAd-SIRPα-Fc, OAd-Siglec10-Fc or OAd-TIGIT-Fc resulted in a robustly increased CD8^+^ T cell/Treg ratio (Fig. 4j and Supplementary Fig. 11).

The changes in TAM (CD11b^+^F4/80^+^Ly6G^−^Ly6C^−^), mMDSC (CD11b^+^Ly6G^−^Ly6C^+^), and gMDSC (CD11b^+^Ly6G^+^Ly6C^−^) infiltration were subsequently analyzed. Intratumoral injections of OAds significantly diminished the tumor infiltration of TAMs in the MC38 and CT26 tumor models (Fig. 4j and Supplementary Fig. 11b). Although the virus injections reduced the percentage of mMDSCs compared with PBS treatment in all three tumor models, the reduction was not associated with “oncolysis” or the secreted fusion proteins (Fig. 4j and Supplementary Fig. 11). However, the percentage of gMDSCs was almost unchanged by the OAds treatments (data not shown). Altogether, these findings demonstrate that intratumoral injections of armed OAds are able to trigger antitumor responses and alter the TME by activating tumor-infiltrating effector T cells and reducing the levels of immunosuppressive cells in tumors.

### Enhanced antitumor activities against distant tumors

**Fig. 5:**
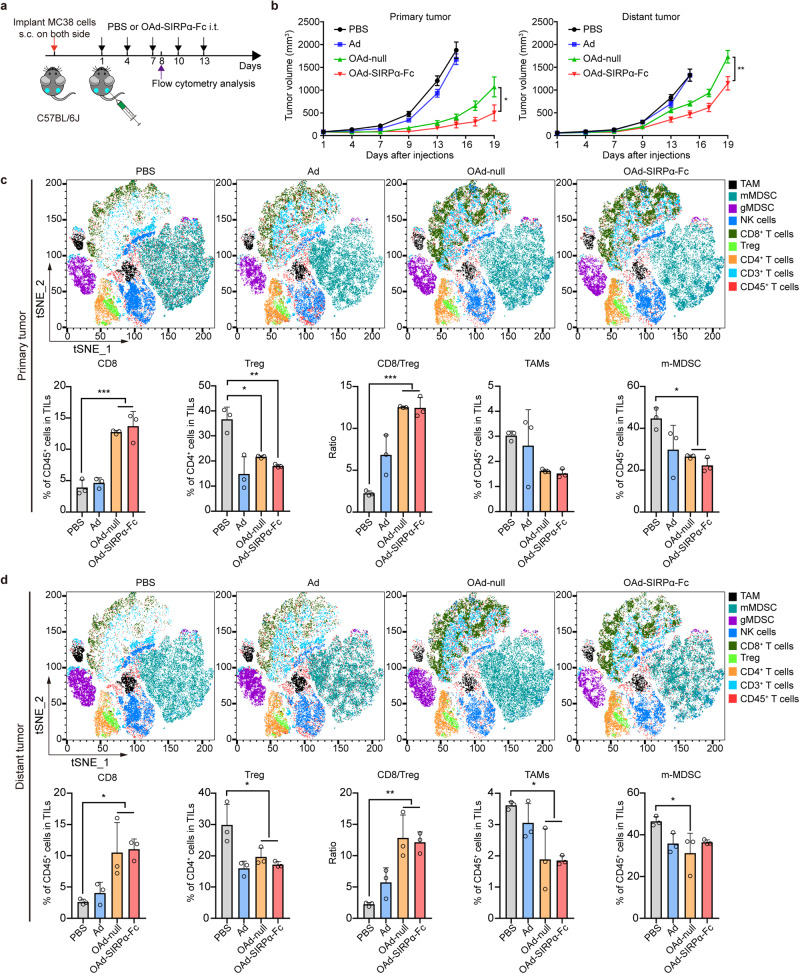
Enhanced antitumor activities against untreated distant MC38 tumors. **a** Mice were subcutaneously inoculated with MC38 tumor cells in both flanks. After establishment of tumors, the right tumor was intratumorally injected with PBS, Ad, OAd-null, or OAd-SIRPα-Fc (1 × 10^8^ pfu per tumor) on days 1, 4, 7, 10, and 13. **b** Growth of injected tumors and distant tumors in the bilateral MC38 tumor model (*n* = 10). Data are represented as mean ± SEM. **c**, **d** TILs in injected tumors (**c**) and distant tumors (**d**) were analyzed by flow cytometry (*n* = 3). Data are represented as mean ± SD. (*p < 0.05, **p < 0.01, ***p < 0.001)

To test whether a localized intratumoral injection of OAds could induce a systemic antitumor immune response, we established bilateral tumor models with MC38, 4T1, or CT26 cells. MC38 tumor-bearing mice had subcutaneous tumors inoculated into both flanks, followed by five injections of OAds into the tumor in the right flank (Fig. 5a). OAd treatment significantly inhibited tumor growth in both the injected and noninjected tumors compared with Ad therapy in the three bilateral tumor models (Fig. 5b; Supplementary Fig. 12a, b and Supplementary Fig. 13a, b).

In the bilateral MC38 tumor model, OAd-SIRPα-Fc treatment achieved greater control of the treated primary tumor and untreated distant tumor than OAd-null therapy (Fig. 5b). Tumor growth suppression of both the treated and contralateral tumors by OAd-Siglec10-Fc was confirmed in 4T1 models (Supplementary Fig. 12a, b). However, there was no difference in tumor inhibition in the CT26 bilateral tumor model between the OAd-null and OAd-TIGIT-Fc treatments (Supplementary Fig. 13a, b). Interestingly, a complete response (CR) of the primary CT26 tumor was observed in 2 of 8 (25%) mice given OAd-null treatment and in 4 of 8 (50%) mice given OAd-TIGIT-Fc treatment (Supplementary Fig. 13a, b). These results supported the potential efficacy of OAd therapy against distant tumors mediated by activating systemic immunity.

To characterize the immunomodulatory effect of intratumoral OAd therapy, infiltrating lymphocytes were analyzed in both tumors of these three bilateral tumor models after the third treatment. The results showed an increased inflammatory response in the virus-injected tumor, with increased infiltration of CD3^+^ lymphocytes (data not shown). Notably, a significant proportion of CD8^+^ T cells infiltrated injected tumors treated with OAds, resulting in elevated CD8^+^ T cells/Treg ratios in the three tumor models. In the MC38 and 4T1 tumor models, the proportion of CD8^+^ T cells and CD8^+^ T cells/Treg ratios were also increased quite significantly in the distant tumors (Fig. 5c, d; Supplementary Fig. 12c, d and Supplementary Fig. 13c, d). These results suggested that the systemic antitumor immunity induced by oncolytic virotherapy could suppress tumor growth.

### Intratumoral administration of OAd-TIGIT-Fc induces the establishment of long-term antitumor memory

**Fig. 6:**
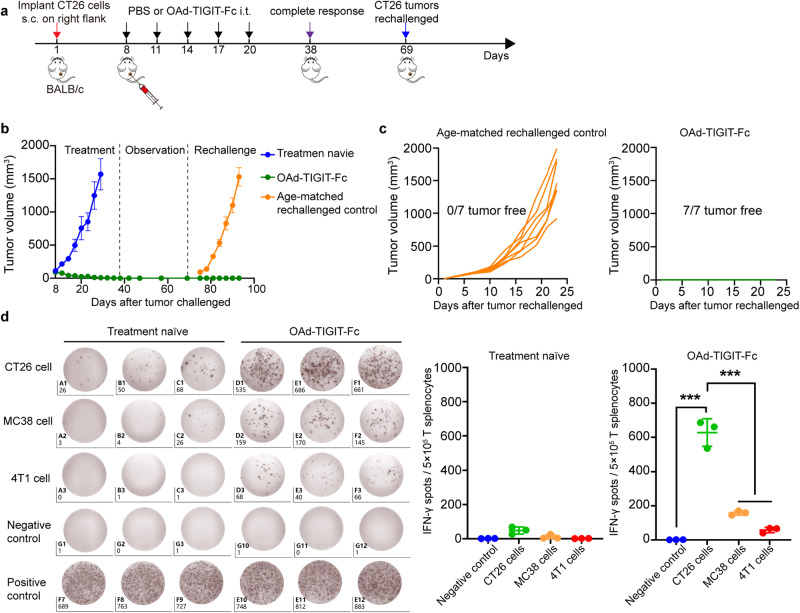
Intratumoral administration of OAd-TIGIT-Fc induces antitumor memory. **a**, **b** BALB/c mice were subcutaneously challenged with 1.5 × 10^6^ CT26 cells (counted as day 1). When tumor sizes reached ~100 mm^3^, the mice were intratumorally injected with 50 μL of OAd-TIGIT-Fc (1 × 10^8^ pfu per tumor) on days 8, 11, 14, 17, and 20. On day 38, seven OAd-TIGIT-Fc-treated mice achieved a CR. On day 69, age-matched naive mice (*n* = 7) and mice with a CR (*n* = 7) were rechallenged with CT26 tumor cells (**b**). Data are represented as mean ± SEM. Tumor growth for individual mice is shown (**c**). **d** Splenic T cells were collected from treatment-naive mice or OAd-TIGIT-Fc-treated mice 45 days after CR achievement to analyze the secretion of IFN-γ in response to CT26 cells in an ELISpot assay (*n* = 3). Data are represented as mean ± SD. (***p < 0.001)

Generation of immune memory is critical for sustained antitumor immunity.^30^ To assess the immune memory triggering potential of intratumoral administration of OAd-TIGIT-Fc, mice were first challenged with CT26 tumor cells and received OAd-TIGIT-Fc treatment. Then, the mice that achieved a CR after OAd-TIGIT-Fc treatment and age-matched naive mice were rechallenged with CT26 tumor cells 69 days after the first challenge. All age-matched control mice developed tumors that grew quickly, whereas all mice with a CR showed no occurrence of a secondary tumor within 25 days of rechallenge (Fig. 6a–c). This result suggested that long-term antitumor memory was established in mice with CT26 tumors cured by OAd-TIGIT-Fc treatment.

To further verify the induction of antitumor memory by OAd-TIGIT-Fc therapy, splenic T cells from OAd-TIGIT-Fc-treated mice with a CR were collected 45 days after CR achievement to analyze the immune response against CT26 cells with an IFN-γ enzyme-linked immunosorbent spot (ELISpot) assay. The number of IFN-γ-secreting cells following stimulation with CT26 cells was significantly greater than that following stimulation with MC38 or 4T1 cells, whereas few splenic T cells from treatment-naive mice exhibited IFN-γ secretion (Fig. 6d). These data indicated that OAd therapy accelerated the generation of long-term antitumor memory.

### Combination immunotherapy with anti-PD-1 and OAd-Siglec10-Fc enhanced tumor regression in the 4T1 model

**Fig. 7:**
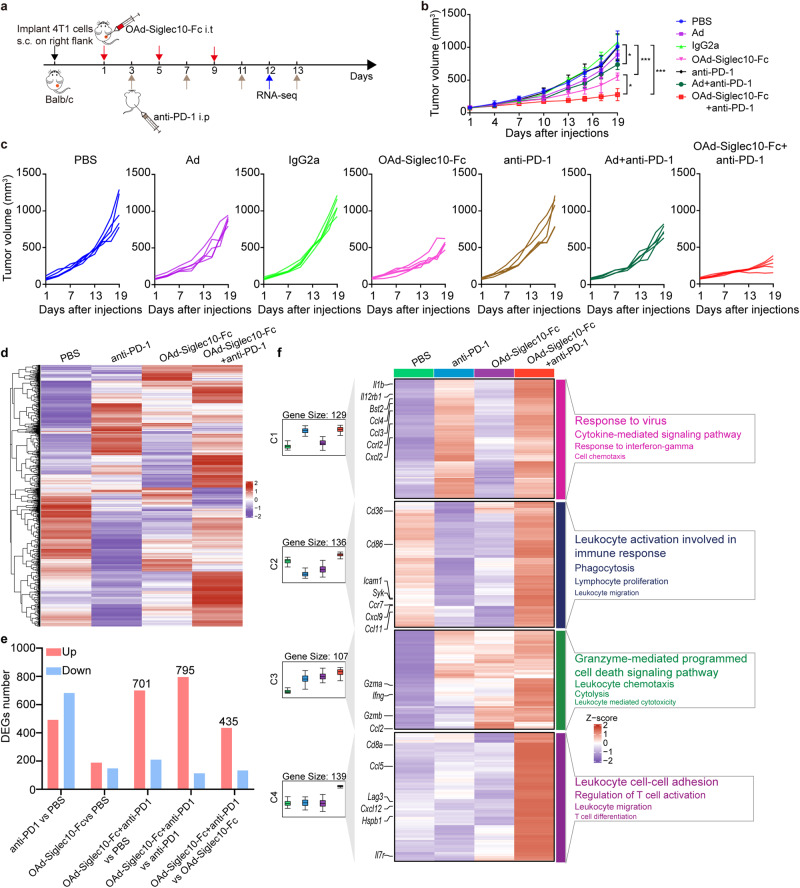
Enhanced antitumor effect of combination therapy with anti-PD-1 and OAd-Siglec10-Fc in the 4T1 model. **a** The treatment schedule for combination therapy with anti-PD-1 and OAd-Siglec10-Fc in the 4T1 model. BALB/c mice were subcutaneously challenged with 1 × 10^6^ 4T1 cells. When tumor sizes reached ~100 mm^3^, the mice were intratumorally injected with 50 μL of OAds (3 × 10^8^ pfu per tumor) on days 1, 5, and 9 and intraperitoneally injected with 200 μg IgG2a or anti-PD-1 on days 3, 7, 11, and 13. Tumor volumes were monitored every three days (**b**), and tumor growth for individual mice is shown (**c**) (*n* = 5). Data are represented as mean ± SD. **d** A heatmap showing all upregulated and downregulated genes among the four groups determined by pairwise comparison. **e** Histogram showing the number of DEGs. **f** The enriched immune response pathways (*n* = 3). (*p < 0.05, ***p < 0.001)

Although OAd-Siglec10-Fc could successfully inhibit the growth of immune “cold” 4T1 tumors by targeting TAMs, further treatment is needed to improve efficacy. As OAd-Siglec10-Fc treatment significantly increased intratumoral CD8^+^ T cell levels, the antitumor effect elicited by the combination of OAd-Siglec10-Fc and PD-1 blockade was investigated further in the 4T1 model. Mice received three intratumoral injections of OAds or PBS on days 1, 5, and 9 and were intraperitoneally injected with IgG2a or anti-PD-1 on days 3, 7, 11, and 13 (Fig. 7a). 4T1 tumor-bearing mice exhibited almost no response to anti-PD-1 blockade (Fig. 7b, c). Combination therapy with OAd-Siglec10-Fc and anti-PD-1 significantly suppressed the growth of 4T1 tumors compared with OAd-Siglec10-Fc or anti-PD-1 monotherapy (Fig. 7b, c).

We next performed transcriptomic analysis to systematically identify the changes in the gene expression of key molecules after combination therapy. Tumor samples from the PBS, anti-PD-1, OAd-Siglec10-Fc and combination therapy groups collected on day 12 were subjected to RNA-seq. All the differentially expressed genes (DEGs) among the four groups identified by pairwise comparison were analyzed. We observed a tendency for drastic upregulation in the combination therapy group compared to the PBS, anti-PD-1 and OAd-Siglec10-Fc groups (Fig. 7d), with 701, 795, and 435 upregulated genes, respectively (Fig. 7e). For functional analysis, we performed GO enrichment analysis of the upregulated genes in the combination therapy group compared with the PBS, anti-PD-1, and OAd-Siglec10-Fc groups (Fig. 7f). There was significant enrichment in immune-related genes in addition to pathways related to leukocyte migration, leukocyte activation involved in the immune response, lymphocyte proliferation, T-cell differentiation, leukocyte-mediated cytotoxicity, cytokine-mediated signaling, etc. (Fig. 7f). We also selected a few important genes encoding chemokines, chemokine receptors, cytokines, Ifng, interleukin costimulatory molecules, and clusters of differentiation and presented the individual gene expression in heatmaps (Supplementary Fig. 14). This suggested that combination immunotherapy with anti-PD-1 and OAd-Siglec10-Fc significantly enhanced the suppression of tumor growth by activating multiple immune signaling pathways in the 4T1 tumor model.

## Discussion

Many attempts have been made to treat cancers by developing multiple immunotherapeutic approaches. The approved anti-CTLA-4, anti-PD-1, and anti-PD-L1 antibodies have become standard treatment options for various solid tumors due to their durable clinical benefit. The main problem with ICI therapy is that its clinical efficacy is limited to a small fraction of patients due to the complexity and uniqueness of the immune microenvironment. The immune microenvironment is highly heterogeneous and unique to each tumor type, and different mechanisms of resistance to ICI therapy, including tumor cell-intrinsic (PD-L1 expression, mutational burden, neoantigen expression, epigenetic variations, interferon-γ signaling, and antigen presentation pathways) and tumor cell-extrinsic (microbiome, PD-L1 expression on immune cells, tumoral and peripheral immune cell composition) mechanisms, have been explored.^23,31^

The limited efficacy of ICI therapy impelled the study of the mechanism of resistance and approaches for rational application in the clinic. Interactions between tumor cells and the immune microenvironment guide the composition and phenotypes of immune subpopulations, which leads to therapeutic resistance to ICIs.^31,32^ In-depth single-cell and transcriptional analyses have been used to profile the immune landscape of massive tumor tissues, including renal cell carcinoma (RCC),^33^ hepatocellular carcinoma (HCC),^34^ non-small cell lung carcinoma (NSCLC),^7^ melanoma, lymphoma^35^ and common central nervous system (CNS) malignancies,^36–38^ demonstrating that the composition and phenotypic state of tumor-infiltrating immune cells vary considerably across tumor types.^5^ Targeting the dominant immune cell population or specific immune cells can increase the response rate. Goswami et al. profiled the immune cell landscape of five different tumor types by mass cytometry.^39^ NSCLC, RCC and CRC tumors were strongly dominated by CD3^+^ T cells, while both prostate cancer (PCa) and glioblastoma multiforme (GBM) had a low frequency of CD3^+^ T cells. Human GBM tumors had strong immune infiltration of CD73^+^ macrophages that persisted after anti-PD-1 treatment, which likely contributed to resistance to ICIs. GBM-bearing mice obtained a survival benefit from the absence of CD73 after treatment with anti-CTLA-4 and anti-PD-1.^39^ In this study, we identified tumor-infiltrating leukocyte phenotypes across four human tumors, glioma, colon cancer, breast cancer and lung cancer, by mIHC. Although all four types of tumors were dominated by MDSCs, glioma, colon cancer and breast cancer also had a high proportion of macrophages, while lung cancer was also dominated by CD8^+^ T cells. Tumor-specific targeting of infiltrating immune cells is a rational strategy for cancer treatment. Thus, identifying the composition and phenotypic states of intratumoral immune cells in different tumor types is critical to developing a tumor-specific ICI strategy for increasing the clinical response rate.

ICIs mainly target T-cell-mediated immune dysfunction to restore the tumor-killing activity of CD8^+^ T cells.^40^ In addition to CTLA-4 and PD-1/PD-L1, there are several second-generation immune checkpoint receptors on T cells, including TIM3, LAG3, TIGIT, CD39, CD73 and VISTA, all of which have been viewed as promising targets for cancer therapy in clinical trials.^41^ TIGIT is expressed on Tregs, memory T cells, activated T cells, and NK cells and binds to CD155 (PVR) and CD112 (PVRL2) on antigen-presenting cells and tumor cells to inhibit T-cell activity and NK cell-mediated cytotoxicity.^42,43^ RNA-seq data from TCGA revealed high expression of CD155 in multiple tumors (Fig. 1c). Patients with low CD155 expression exhibited an OS advantage compared to patients with high CD155 expression (Fig. 1c, d). Blockade of TIGIT with an antibody or fusion protein effectively restores CD8^+^ T cell antitumor immunity^44–46^ and prevents NK cell exhaustion.^47^ Macrophages play a vital role in engulfing cancer cells and processing antigens for presentation to stimulate adaptive immunity.^48^ TAMs, a subset of macrophages that are abundant within the TME,^49^ lose the ability to phagocytize tumor cells via the interactions of immunosuppressive receptors with ligands expressed by tumor cells, such as CD47-SIRPα signaling^50^ and CD24-Siglec10 signaling,^11^ and this effect is especially prominent in M2 TAMs.^51^ High expression of CD47 and CD24 was observed in multiple types of human tumors, corresponding with impaired OS benefits in patients with high expression compared to those with low CD47 and CD24 expression (Fig. 1c, d). Targeting the “do not eat me” signals CD47 and CD24 with an antibody or Fc-fusion proteins increases the macrophage phagocytosis of tumor cells and enhances antitumor immunity.^11,52^

OVs play an important role in the field of tumor therapy through the dual functions of exerting “oncolytic” activity and activating the body’s antitumor immune response. Here, we analyzed the composition of intratumoral immune cells and identified the expression of immune checkpoint ligands. Then, three-armed cancer-targeting OAds were developed: OAd-SIRPα-Fc and OAd-Siglec10-Fc, which expressed SIRPα-Fc or Siglec10-Fc, respectively, to target macrophages to restore the phagocytic capabilities of these cells, and OAd-TIGIT-Fc, which secreted TIGIT-Fc to reactivate T cells (Fig. 2a). The results of this study demonstrated that OAd-SIRPα-Fc, OAd-Siglec10-Fc and OAd-TIGIT-Fc were able to produce SIRPα-Fc, Siglec10-Fc and TIGIT-Fc, respectively, which effectively bound to CD47^+^, CD24^+^ and CD155^+^ tumor cells (Fig. 2e). The engineered OAd-SIRPα-Fc and OAd-Siglec10-Fc generated in this study showed outstanding efficacy in tumor suppression in macrophage-dominated tumors (Fig. 3b). It is postulated that the secreted SIRPα-Fc and Siglec10-Fc blocked the corresponding “do not eat me” signals and thus restored TAM‐mediated tumor cell phagocytosis. OAd-SIRPα-Fc and OAd-Siglec10-Fc treatments had higher C1qc signature scores than OAd-null therapy (Supplementary Fig. 7a). C1qc^+^ TAMs show preferential expression of genes related to phagocytosis and antigen presentation, while Spp1^+^ TAMs are enriched for regulators of angiogenesis and have a pro-angiogenic signature.^53^ Moreover, OAd-SIRPα-Fc treatment increased the proportion of TAM-C2, which had high C1qc signature scores (Fig. 3e, h) and showed significant enrichment of gene expression signatures in lysosomal and phagosomal pathways and in the proinflammatory phenotype, such as antigen presentation pathways, response to TNF, and IL17 signaling.^54^ OAd-TIGIT-Fc showed the best induction of antitumor immunity in CD8^+^ T cell-dominated tumors, with a high cytotoxicity score and the lowest exhaustion score.^44^

Beyond the delivery of immunoregulatory genes (such as chemokines, cytokines, T-cell costimulatory molecules, antigens, ICIs, etc.), OVs can lyse tumor cells specifically and activate innate immunity and antitumor adaptive immunity.^13^ In recent years, multiple OVs have been approved to offer safer and more effective alternative therapies for patients with refractory cancers.^55–57^ Several observations imply that the presence of T cells within the TME has been viewed as a marker for ICI therapy.^58,59^ Oncolytic virotherapy could effectively promote the intratumoral infiltration of T cells for cancer therapy or combination with ICI therapy.^60,61^ Our nonclinical data showed that intratumoral administration of an OAd significantly enhanced CD8^+^ T cell infiltration and decreased the proportions of suppressive immune cells, such as mMDSCs, TAMs and Tregs (Fig. 4j and Supplementary Fig. 11a, b). The Fc-fusion proteins expressed by the OAds further improved the activation of immune responses and the inhibition of immunosuppressive responses, which are essential for effective tumor immunotherapy.^62^ The systemic immune response and immune memory play vital roles in durable antitumor immunity.^63,64^ The OAds in this study resulted in a systemic immune response against distant noninjected tumor sites (Fig. 5b; Supplementary Fig. 12a, b and Supplementary Fig. 13a, b) and long-term tumor-specific immune memory against secondary tumor challenges (Fig. 6b, c). Oncolytic virotherapy can reprogram an immunosuppressive TME but can also significantly increase the expression of PD-L1 on breast cancer cells, which leads to immune escape after oncolytic virotherapy.^22^ The combination of an OV and anti-PD-1 can solve this problem perfectly. Talimogene laherparepvec (T-VEC) is a herpes simplex virus armed with granulocyte-macrophage colony-stimulating factor (GM-CSF). However, in a phase III study, T-VEC plus anti-PD-1 did not significantly improve progression-free survival or OS compared with placebo plus anti-PD-1 in patients with advanced melanoma.^65^ This suggests that it is important to design OVs based on the TME. In our study, the treatment of 4T1 tumor models using OAd-Siglec10-Fc combined with anti-PD-1, which targets both TAMs and T cells, was found to be a reasonable and promising strategy that altered the antitumor immune response, promoted phagocytosis by macrophages and enhanced the effects of subsequent treatment with ICIs.

In summary, our data demonstrated that engineered OAds rationally designed based on the understanding of the composition and phenotypic states of intratumoral immune cells not only precisely inhibited tumor growth but also induced systemic immunity and long-term immune memory, providing a tumor-specific ICI-delivering virotherapy strategy. Moreover, ICI-delivering virotherapy could be used as monotherapy or has the potential to be combined with other ICIs, especially approved anti-PD-1/PD-L1 antibodies. Based on this platform, more specific and effective OAds could be designed to offer safer and more effective therapies for patients with refractory cancers.

## Materials and methods

### Cell lines

The human embryonic kidney 293 (HEK293) cell line, HEK293A cell line, murine breast cancer cell line 4T1, murine colon cancer cell line CT26, murine glioma cell line GL261, murine lung carcinoma cell line LL2 and mouse embryo fibroblast cell line 3T3-L1 were purchased from the American Type Culture Collection (ATCC). The murine colon cancer cell line MC38 was purchased from Kerafast. HEK293, HEK293A, MC38, GL261, LL2, and 3T3-L1 cells were cultured in Dulbecco’s modified Eagle’s medium (4T1 and CT26 cells were cultured in RPMI-1640 medium) supplemented with 10% heat-inactivated fetal bovine serum (Gibco) and 1% penicillin‒streptomycin antibiotic mixture (Sigma Aldrich). All cells were grown in an incubator at 37 °C and 5% CO_2_.

### Recombinant OAd generation and purification

Recombinant adenoviruses OAd-null, OAd-SIRPα-Fc, OAd-Siglec10-Fc, and OAd-TIGIT-Fc were cloned using AdMax system in HEK293 cells. Based on the type 5 adenovirus, an mhTERT promoter to drive the expression of the E1 gene was inserted into the plasmid pDC316 shuttle vector (pDC316-mhTERT), in which a 24-bp sequence in the E1A region and an E1B55-kD viral protein in the E1B region were deleted.^25,26^ Then, the murine soluble SIRPα, Siglec10 or TIGIT extracellular domains fused with IgG1 Fc respectively, driven by the cytomegalovirus (CMV) promoter were inserted into the ∆E3 region of the genomic plasmid pBHGloxΔE1,3Cre with Red/ET recombination. Named pBHGloxΔE1,3Cre-SIRPα-Fc; pBHGloxΔE1,3Cre-Siglec10-Fc; pBHGloxΔE1,3Cre-TIGIT-Fc (Fig. 2a). To generate adenoviruses, pDC316-mhTERT and pBHGloxΔE1,3Cre-SIRPα-Fc; pBHGloxΔE1,3Cre-Siglec10-Fc or pBHGloxΔE1,3Cre-TIGIT-Fc were cotransfected into HEK293 cells. Cells and supernatants were collected one week after transfection and lysed with three consecutive ‘freeze‒thaw’ cycles. Plaques were subjected to two plaque purifications, and the integrity of the E1A region, E2B region and fusion gene in the E3 region was characterized by PCR and confirmed by DNA sequencing. The amplified viruses were centrifuged with CsCl gradients (Sigma Aldrich) and purified with dialysate (10 mM Tris-HCl (pH = 8.0), 2 mM MgCl_2_, 50 mM NaCl, 4% sucrose). The viruses were stored at −80 °C for future use in animal studies.

### Viral titer determination

HEK 293 A cells (1 × 10^4^) were seeded in 96-well microtitration plates, serial 10-fold dilutions of virus supernatant were made, and 100 μL of each dilution was inoculated into 10 wells of the 96-well microtitration plates the next day. The plates were incubated for 10 days in an incubator at 37 °C and 5% CO_2,_ and then the cultures were checked under a light microscope for cytopathology. Viral titers were determined as the median tissue culture infectious dose (TCID_50_).

### Transmission electron microscopy observation

A transmission electron microscope (HITACHI, Tokyo, Japan) at an acceleration voltage of 200 keV was used to observe purified virions. A droplet of virus suspension was placed on a copper grid and stained with 3% phophatungstic acid for 45 s. Filter paper was used to remove excess water from samples, which were then dried at room temperature.

### Detection of mhTERT priming activity using a dual-luciferase reporter assay

Cells (2 × 10^4^) were seeded in 96-well microtitration plates and incubated at 37 °C for 24 h. For each experimental condition, the corresponding transfection mix containing the plasmid DNA (Supplementary Table 3) was prepared with Lip3000 Transfection Reagent (Invitrogen). The wt-hTERT promoter and mhTERT promoter were inserted into the pGL3-basic vector separately to generate reporter vectors containing the firefly luciferase gene. The Dual-Glo^®^ Luciferase Assay System (Promega) was used to measure firefly and *Renilla* luciferase luminescence as the manufacturer described after transfection for 24 h. Each experiment included identical transfections in triplicate for each test group.

### Cell survival experiments

Cells (2 × 10^3^) were seeded in 96-well microtitration plates and incubated at 37 °C for 24 h before infection with the indicated adenoviruses or PBS at various MOIs in triplicate. Cell survival was assessed at 72 h after infection using a Cell Counting Kit-8 assay (MedChemExpress) according to the manufacturer’s instructions. The half-maximal inhibitory concentration (IC_50_) values of the adenoviruses were determined by interpolation from a sigmoidal dose‒response curve fit of the log-transformed survival data using GraphPad Prism software.

### Western blot analysis

Tumor cells (4 × 10^6^) cultured in 10-cm^2^ dishes were infected with the indicated OAds at an MOI of 15. After incubation for 48 h, the supernatants were harvested and clarified by centrifugation at 4000 × *g* for 5 min. Then, 100 μL of protein A was added to the supernatants, and the mixtures were incubated on a rocking shaker at 4 °C for at least 4 h. Then, the pellets were collected by centrifugation at 4000 × *g* for 5 min and washed two times with cold PBS. The pellets were resuspended in 200 μL of PBS and mixed with 4× protein sodium dodecyl sulfate (SDS) sample loading buffer or 4× protein native sample loading buffer (without SDS). After heating in a metal bath at 100 °C for 10 min, the supernatants were collected by centrifugation at 10,000 × *g* for 2 min and electrophoresed in a 10% SDS–polyacrylamide gel (Epizyme Biotech). The separated protein samples were transferred to a 0.45-μm nitrocellulose membrane with eBlot^TM^ L1 (GenScript). The nitrocellulose membrane was blocked in TBST buffer containing 5% nonfat milk for 1 hour on a rocking shaker at room temperature. Immunodetection of SIRPα-Fc, Siglec10-Fc, and TIGIT-Fc was performed by incubation with a horseradish peroxidase (HRP)-conjugated goat anti-mouse IgG antibody (Cell Signaling Technology) at room temperature for 1 h or with rabbit anti-mouse SIRPα-Fc (Thermo Fisher Scientific), Siglec10-Fc (Cell Signaling Technology) and TIGIT-Fc (Abcam) overnight at 4 °C, followed by incubation with HRP-conjugated secondary anti-rabbit IgG (Cell Signaling Technology). Finally, the target protein bands were detected with a chemiluminescence system (Millipore, Massachusetts, USA).

### Fusion protein binding assessment by flow cytometry

shRNA targeting sequences against mouse CD47, CD24, or CD155 (shown in Supplementary Table 4) or scrambled (control) oligonucleotides were annealed and then ligated into the *AgeI* and *EcoRI* sites of the pLKO.1-TRC cloning vector (OligoEngine). Retroviral particles were generated and introduced into MC38, 4T1 or CT26 cells. Infected cells were selected and maintained with an appropriate concentration of puromycin. shCD47/MC38, shCD24/4T1 and shCD155/CT26 cells were assessed by flow cytometry with appropriate antibodies. To detect fusion protein binding, wild-type MC38, 4T1, or CT26 cells and the corresponding ligand-knockdown cells were incubated with purified SIRPα-Fc, Siglec10-Fc and TIGIT-Fc isolated from corresponding virus-infected tumor cells and then stained with a PE/Cyanine7-conjugated anti-mouse IgG1 antibody (BioLegend) for flow cytometry analysis.

### Mouse experiments

All animal procedures were approved and controlled by the Institutional Animal Care and Treatment Committee of Sichuan University and conducted according to the Animal Care and Use Guidelines of Sichuan University. Female C57BL/6J and BALB/c mice (6 to 8 weeks old) were supplied by Charles River (Zhejiang, China) and quarantined for at least 1 week. Chow and drinking water were available to mice ad libitum. The diameters of subcutaneous tumors were measured using a digital caliper, and tumor volume was calculated by the following formula: Volume = 0.5 × length × width^2^. Mice were euthanized when signs of deterioration or acute weight loss were observed or when the tumor size exceeded ~2000 mm^3^. When tumors were not detectable by palpation, mice were defined as having achieved complete tumor regression. The cell line, supplier, genetic strain and sex of the mouse from which each cell line was derived, tissue of origin of the tumor from which each cell line was originally derived, and number of cells injected subcutaneously to form tumors are given in Supplementary Table 5.

To evaluate OAd-null, OAd-SIRPα-Fc, OAd-Siglec10-Fc, and OAd-TIGIT-Fc in immunocompetent mice, unilateral MC38, 4T1 and CT26 models were used. In these models, 1 × 10^6^ MC38 or 4T1 cells or 1.5 × 10^6^ CT26 cells were subcutaneously inoculated into the right flank. When the tumors reached 80–100 mm^3^, the mice were divided randomly into treatment groups (*n* = 5 or 10 per group). For tumor growth studies and survival studies using tumor-bearing mice, 50 μL of 1 × 10^8^ pfu or 3 × 10^8^ pfu of the indicated adenoviruses or phosphate-buffered saline (PBS) was injected into individual tumors every three days for a total of five injections. To evaluate the TME, tumors were harvested after the third injection. For MC38 and CT26 tumor tissues, single-cell sequencing was performed according to the methods described below. Flow cytometry was also used to detect the infiltration of immune cells in MC38, 4T1, and CT26 tumor tissues.

To examine the abscopal effects of OAd-SIRPα-Fc, OAd-Siglec10-Fc, and OAd-TIGIT-Fc, bilateral MC38, 4T1, and CT26 models were used. A total of 1 × 10^6^ MC38 or 4T1 cells or 1.5 × 10^6^ CT26 cells were subcutaneously inoculated into the right flank, and half that number of tumor cells was inoculated into the left flank at the same time. When the tumors on the right flank reached 80–100 mm^3^, unilateral intratumoral treatment was commenced as described above. The TME of each side was detected by flow cytometry after three injections of the corresponding adenovirus.

In rechallenge studies, BALB/c mice with CT26 tumors that had been cured by OAd-TIGIT-Fc treatment and age-matched treatment-naive mice were subcutaneously inoculated with 1.5 × 10^6^ CT26 cells in the right flank.

### cDNA microarray analysis

Human cancer tissue cDNA microarrays of colon adenocarcinoma (*n* = 30), liver hepatocellular carcinoma (*n* = 30), lung adenocarcinoma (*n* = 15) and stomach adenocarcinoma (*n* = 30) were purchased from Shanghai Outdo Biotech. The mRNA expression of CD24, CD47 and CD155 in the cancer samples in these cDNA microarrays was detected using SYBR Green by quantitative real-time PCR on an Applied Biosystems QuantStudio 3 Real-Time PCR System (Thermo Fisher, USA). The sequences of the PCR primers were as follows: CD24 forward: 5′-CCTACCCACGCAGATTTATT-3′, reverse 5′-TGGTGGCATTAGTTGGATTT-3′; CD47 forward: 5′-AGAAGGTGAAACGATCATCGAGC-3′, reverse 5′-CTCATCCA TACCACCGGATCT-3′; CD155 forward: 5′-TGGAGGTGACGCATGTGTC-3′, reverse 5′-GTTTGGACTCCGAATAGCTGG-3′; GAPDH forward: 5′-GGAGCGAG ATCCCTCCAAAAT-3′, reverse 5′- GGCTGTTGTCATACTTCTCATGG-3′. GAPDH served as the endogenous control gene.

### Single-cell transcriptomic analysis

MC38 and CT26 tumors were harvested from mice after three injections of the indicated adenovirus or PBS. To obtain single-cell suspensions, the tumors were mechanically disrupted prior to enzymatic digestion with the GentleMACS Mouse Tumor Dissociation kit (Miltenyi Biotech) according to the manufacturer’s instructions. The digested tissues were then passed through a 40-μm Cell-Strainer and centrifuged at 300 × *g* for 7 min. Next, red blood cell lysis was performed with red blood cell lysis buffer on ice. After washing twice with PBS, the single-cell suspensions were resuspended in RPMI-1640 medium. Dead cells were eliminated by labeling the cells with Dead Cell Removal MicroBeads (Miltenyi Biotech) and separating them over an LS Column in the magnetic field of a MidiMACS Separator. The samples were subjected to library preparation and scRNA-seq using the 10× Genomics protocol for the Illumina NovaSeq 6000 sequencing platform.

All analyses were carried out in the NIH Biowulf high-performance computing environment. We used the following R packages for analyses: Seurat_4.1.1, stats_3.6.3, GSVA_1.40.1, msigdbr_7.5.1, limma_3.48.3, clusterProfiler_4.0.5, CellChat_1.4.0, and ComplexHeatmap_2.8.0. The software Cell Ranger provided by 10× Genomics was applied to align reads and generate a gene-cell unique molecular identifier (UMI) matrix using the reference genome GRCm38. For each cell, we quantified the numbers of genes and UMIs and kept high-quality cells with a detection threshold of 600–6000 genes and 1600–35,000 UMIs to filter out most of the barcodes associated with debris or cell doublets. Cells with an unusually high detection rate of mitochondrial gene expression (>10%) were also excluded as described above.

The Seurat package was used for clustering and uniform manifold approximation and projection (UMAP) analysis. Clustering analysis was performed with FindClusters. Run-UMAP was used to visualize samples. FindMarkers was used to identify DEGs with the Wilcox test, and adjusted *P* values were computed by using the BH test implemented in the stats function *p*.adjust. DEGs were defined as follows: absolute log2-fold change >0.5 and adjusted *P* value of BH test <0.05.

Gene set variation analysis implemented with the GSVA package (version 1.40.1)^66^ was used for gene set enrichment analysis. The KEGG pathway gene sets were exported by using the msigdbr package (version 7.5.1). The activities of pathways between cells in different groups were scored with the limma package (version 3.48.3).^67^ KEGG enrichment analysis was performed by clusterProfiler (version 4.0.5).^68^

To evaluate the biological functions of cell clusters in different immune cell types, we used the *Seurat* function *AddModuleScore* to define the M1 score for macrophage cluster based on “classically activated” (M1) macrophage-related genes, the M2 score for macrophage clusters based on “alternatively activated” (M2) macrophage-related genes, the C1qc^+^ score for macrophage clusters based on C1qc^+^ TAM gene signatures, the Spp1^+^ score for macrophage clusters based on Spp1^+^ TAM gene signatures, the cytotoxicity score for T cell and NK clusters based on cytotoxicity-associated genes, the exhaustion score for T cell and NK clusters based on exhaustion-associated genes and the naive score for T cell and NK clusters based on naive markers. The related genes for scoring are shown in Supplementary Table 2.

The CellChat package (version 1.4.0) and CellChat database were used to analyze and infer cell‒cell communication.^69^

All statistical analyses were performed using R (version 4.0.3). All figures were plotted by using R. *P* < 0.05 or *P* adjust <0.05 were considered statistically significant.

### Flow cytometric analysis of the TME

To analyze tumor-infiltrating lymphocytes, tumors were isolated from mice when the tumor volume reached 80–100 mm^3^ or after three treatments with the indicated adenoviruses or PBS. Single-cell suspensions of tumor cells were obtained as described above and stained with Fixable Viability Stain 700 (BD Biosciences) for 10 min, followed by staining with a surface antibody cocktail prepared in brilliant stain buffer (BD Biosciences) at 4 °C for 30 min in the dark. The following fluorophore-conjugated anti-mouse antibodies were used: anti-CD45-BUV395 (clone 30-F11, BD Biosciences), anti-CD3-APC-Cy™7 (clone 145-2C11, BD Biosciences), anti-CD4-BV510 (clone RM4-5, BD Biosciences), anti-CD8-PerCP-Cy™5.5 (clone 53-6.7, BD Biosciences), anti-CD335-BV421 (clone 29A1.4, BD Biosciences), anti-CD11b-FITC (clone M1/70, BD Biosciences), anti-Ly6G-PE-Cy™7 (clone 1A8, BD Biosciences), anti-Ly6C-BV605 (clone AL-21, BD Biosciences), anti-CD86-BV786 (clone GL1, BD Biosciences) and anti-F4/80-PE (clone T45-2342, BD Biosciences). Intracellular staining was performed using the transcription factor buffer kit (BD Biosciences) according to the manufacturer’s instructions after surface staining and involved staining with anti-CD206-BV650 (clone C068C2, BioLegend) and anti-FoxP3-Alexa Fluor^®^ 647 (clone MF23, BD Biosciences) at 4 °C for 50 min in the dark.

Samples were acquired on a flow cytometer (BD Biosciences, FACSymphony A5), and FACS data were processed by using FlowJo software (v.10). Cell populations were defined as indicated in Supplementary Fig. 10.

### mIHC

To investigate the cellular composition of spatially distinct tumors, tumor microarrays were obtained from Outdo Biotech Co., Ltd. (Shanghai, China) and mIHC was performed as described in the literature.^70^ After deparaffinization, hydration and antigen unmasking, the slides were quenched with 3% hydrogen peroxide. Then, a primary antibody was added to each section flowed by boost IHC detection reagent (Cell Signaling Technology) specific to the species of the primary antibody. Then, the fluorophore-conjugated Tyramide Signal Amplification (TSA) plus amplification reagent (Akoya Biosciences) was used and stripped with 1 mM EDTA (pH 8.0). Then, the next primary antibody was added, and this procedure was repeated until a total of 6 antibodies were finished. Finally, the slides were incubated with Opal DAPI and mounted with coverslips using prolonged gold antifade reagent with DAPI (Cell Signaling Technology). The multiplex panel used in this article is shown in Supplementary Table 6. The PerkinElmer Vectra3^®^ platform was used to scan and image slides. Immunocytes were selected and batch analyzed using PerkinElmer Inform software.

### TUNEL assay

After the antitumor test experiment was completed, tumor samples were harvested, fixed with 4% paraformaldehyde and embedded in paraffin. Apoptotic cells with DNA fragmentation were detected in the embedded tissue sections using the TUNEL Bright Red Apoptosis Detection Kit (Vazyme Biotech) following the manufacturer’s protocol.

### Ex vivo analysis of immune responses

A mouse IFN-γ precoated ELISpot kit (DAKEWE) was used to detect immune responses. According to the manufacturer’s protocol, spleens were collected from treatment-naive mice and mice that had previously been cured of CT26 tumors by OAd-TIGIT-Fc treatment. Lymphocytes were isolated and cultured with irradiated (100 Gy) CT26, MC38 or 4T1 cells at a ratio of 50:1 (total cell number: 1 × 10^5^ cell/well) in ELISpot plates precoated with an anti-IFN-γ antibody. The plate was incubated at 37 °C with 5% CO_2_ for 48 h, and then precooled ddH_2_O was added and incubated at 4 °C for 10 min to lyse the cells. After washing five times with wash buffer, a diluted biotinylated secondary antibody was added to each well, followed by incubation for 1 h at 37 °C. For enzyme-linked avidin incubation, a diluted avidin enzyme working solution was added to each well and incubated at 37 °C for 1 h. A prepared aminoethyl carbazole solution was then added, and the color reaction was allowed to occur at 37 °C in the dark for approximately 10 min. Finally, the plates were photographed and read using a BioReader 4000 (Byosys, Karben, Germany).

### RNA library construction and data analysis

Flash-frozen tumors (*n* = 3 mice per treatment group) were pulverized, and total RNA was extracted using the RNeasy Mini Qiacube extraction kit according to the manufacturer’s protocol. Poly(A)-tailed mRNA was enriched, and an RNA-seq library was constructed by the NEBNext^®^ Ultra™ RNA Library Prep Kit for Illumina^®^ following the manufacturer’s instructions. RNA-seq data were generated on an Illumina NovaSeq 6000 using the 150-bp pair-ended running mode. After removing reads with sequencing adaptors, unknown reads with “N” and low-quality reads, clean reads were mapped to a reference genome. Differential expression was analyzed using DESeq2 software. A heatmap of the log2(fold change) expression of DEGs was drawn with pheatmap, and GO enrichment analysis of DEGs was performed by Phyper based on the hypergeometric test.

### Statistics

The significance of differences between two groups was determined using Student’s t test, and one-way ANOVA was used for multiple-group comparisons. Statistical analysis was performed using GraphPad Prism 7 software. A *p* value of less than 0.05 was considered statistically significant.

### Supplementary information

Supplementary information-Clean

## Data Availability

The raw scRNA-seq data of MC38 and CT26 tumor samples and the raw RNA-seq data of 4T1 tumor samples have been deposited in the Genome Sequence Archive (Genomics, Proteomics & Bioinformatics 2021) in National Genomics Data Center (Nucleic Acids Res 2022), China National Center for Bioinformation/Beijing Institute of Genomics, Chinese Academy of Sciences (GSA: CRA012704, GSA: CRA012707, GSA: CRA012733) that are publicly accessible at https://ngdc.cncb.ac.cn/gsa. The datasets generated and/or analyzed during the current study are available from the corresponding author upon reasonable request.
