# Supplementary material for: Oncolytic adenoviruses expressing checkpoint inhibitors for cancer therapy
Source: Signal Transduct Target Ther. 2023 Nov 29;8:436. doi: 10.1038/s41392-023-01683-2 (PMC10684539; doi:10.1038/s41392-023-01683-2)
Supplement: Supplementary file 1 — Supplementary information-Clean [file 41392_2023_1683_MOESM1_ESM.docx]

Supplementary Materials for

Oncolytic adenoviruses expressing checkpoint inhibitors for cancer therapy

Daoyuan Xie^1^, Yaomei Tian^1, 2^, Die Hu^1^, Yuanda Wang^1^, Yuling Yang^1^, Bailing Zhou^1^, Rui Zhang^1^, Zhixiang Ren^1^, Mohan Liu^1^, Jie Xu^1^, Chunyan Dong^1^, Binyan Zhao^1^, and Li Yang^1^

Correspondence to: yl.tracy73@gmail.com

**This PDF file includes:**

Supplementary Results

Figures. S1 to S14

Tables S1 to S6

Supplementary Results

scRNA-seq analysis of the CT26 tumor model

In the CT26 tumors, after unbiased cell-type classifications using Seurat v4, 7 cell clusters were identified based on marker gene expression, including cDCs by *H2-Aa*, *H2-Eb1*, *H2-Ab1*, *CD74* and *H2-DMb2*; pDCs by *Klk1*, *Siglech*, *Cox6a2*, *Rnase6* and *Irf8*; macrophages by *Lyz2, Apoe, Fcer1g, CD68* and *Msr1*; neutrophils by *Cxcl2*, *Il1b*, *CCrl2*, *Srgn* and *Ccl3*; NK cells by *Gzma*, *Il2rb*, *prf1*, *Nkg7* and *Ncr1*; and T cells by *CD3e*, *CD3g*, *Ms4a4b*, *CD3d* and *Gzmb* (Supplementary Fig. 8a and Supplementary Fig. 9). Compared to PBS tumors, we observed increased proportions of T cells in the other four OAds treatments, especially in the OAd-Siglec10-Fc and OAd-TIGIT-Fc groups (Supplementary Fig. 8b). The level of macrophages was obviously increased by OAd-TIGIT-Fc treatment (Supplementary Fig. 8b). We calculated M1^+^ and M2^+^ scores for macrophage cluster by using the *Seurat* function *AddModuleScore* to analyze the functional states (Supplementary Fig. 8c). Compared with PBS, OAds therapies exhibited significantly higher M1^+^ scores but no significant difference in M2^+^ scores. OAd-SIRPα-Fc and OAd-TIGIT-Fc showed higher M1^+^ scores than OAd. We did not find a significant difference in the M1^+^ scores between OAd-null and OAd-Siglec10-Fc groups (Supplementary Fig. 8c). Furthermore, OAds treatments showed higher cytotoxicity scores for T cells than PBS (Supplementary Fig. 8d). OAd-SIRPα-Fc and OAd-TIGIT-Fc showed lower exhaustion scores for T cells than PBS and OAd-null (Supplementary Fig. 8d).

Based on the expression of canonical markers, we annotated macrophages into seven subtypes (TAM-C1 to TAM-C7) (Supplementary Fig. 8e). Compared with PBS, the proportions of TAM-C2 and C7 were increased and those of TAM-C1 and C5 were markedly decreased in OAds treatments, especially OAd-TIGIT-Fc (Supplementary Fig. 8f). To better understand the roles of these populations, we further calculated M1 and M2 gene signatures (Supplementary Fig. 8g). Although the M1 and M2 scores of macrophage subclusters C1, C2, C5 and C7 had the same changing tendency, significant enrichment of gene expression signatures in the proinflammatory phenotype, such as antigen processing and presentation, HIF-1 signaling, TNF-α signaling, NF-κB signaling and chemokine signaling, was observed in TAM-C2 and TAM-C7 (Supplementary Fig. 8h). Importantly, our data show that the lysosome and phagosome pathways of TAM-C2 and TAM-C7 were stronger than those of TAM-C1 and TAM-C5, which possessed the characteristics of M2 macrophages via enrichment of gene expression signatures of oxidative phosphorylation.

We further performed unsupervised clustering of T cells and obtained 12 clusters: CD3-C1; CD4-C1, CD4-C2; CD8-C1 to CD8-C7 (Supplementary Fig. 8i). Compared to PBS, the abundance of CD4-C2, CD8-C5 and CD8-C7 was decreased in the OAds treatments. However, the relative percentages of CD8-C2 and CD8-C3 in the OAds treatment groups were significantly increased compared with those in the PBS group (Supplementary Fig. 8j). Based on the expression of canonical markers (Supplementary Fig. 8k), we identified Tregs (CD4-C2: *Foxp3^+^*, *TIGIT^+^ and CTLA4^+^*), TRM/TEM T cells (CD8-C5: *INFg^+^*, *Ccl4^+^ and Ccl3^+^*) and terminal exhausted T cells (CD8-C7: *PDCD1^+^*, *LAG3^+^*, *HAVCR2^+^* and *CD96^+^*). Importantly, CD8-C2 and CD8-C3 showed high expression levels of *GZMA*, *GZMK* and *CCL5*, representing cytotoxic T cells with a high cytotoxicity T cell gene signature score but a low exhausted T cell gene signature score (Supplementary Fig. 8l). However, CD8-C3 exhibited a high level of exhaustion compared to CD8-C2. Treg cells (CD4-C2) exhibited a low cytotoxicity score but a high exhaustion score. CD8-C5 only exhibited a high exhaustion score. CD8-C7 showed high cytotoxicity and exhaustion scores, indicating an activation-coupled exhaustion program. Notably, that OAd-TIGIT-Fc treatment resulted in a high proportion of CD8-C2 and a low percentage of CD8-C7 compared to the other groups. These data demonstrated that OAd-TIGIT-Fc treatment mainly enhanced the activation of cytotoxic CD8^+^ T cells, and alleviated immunosuppression in the CT26 model.


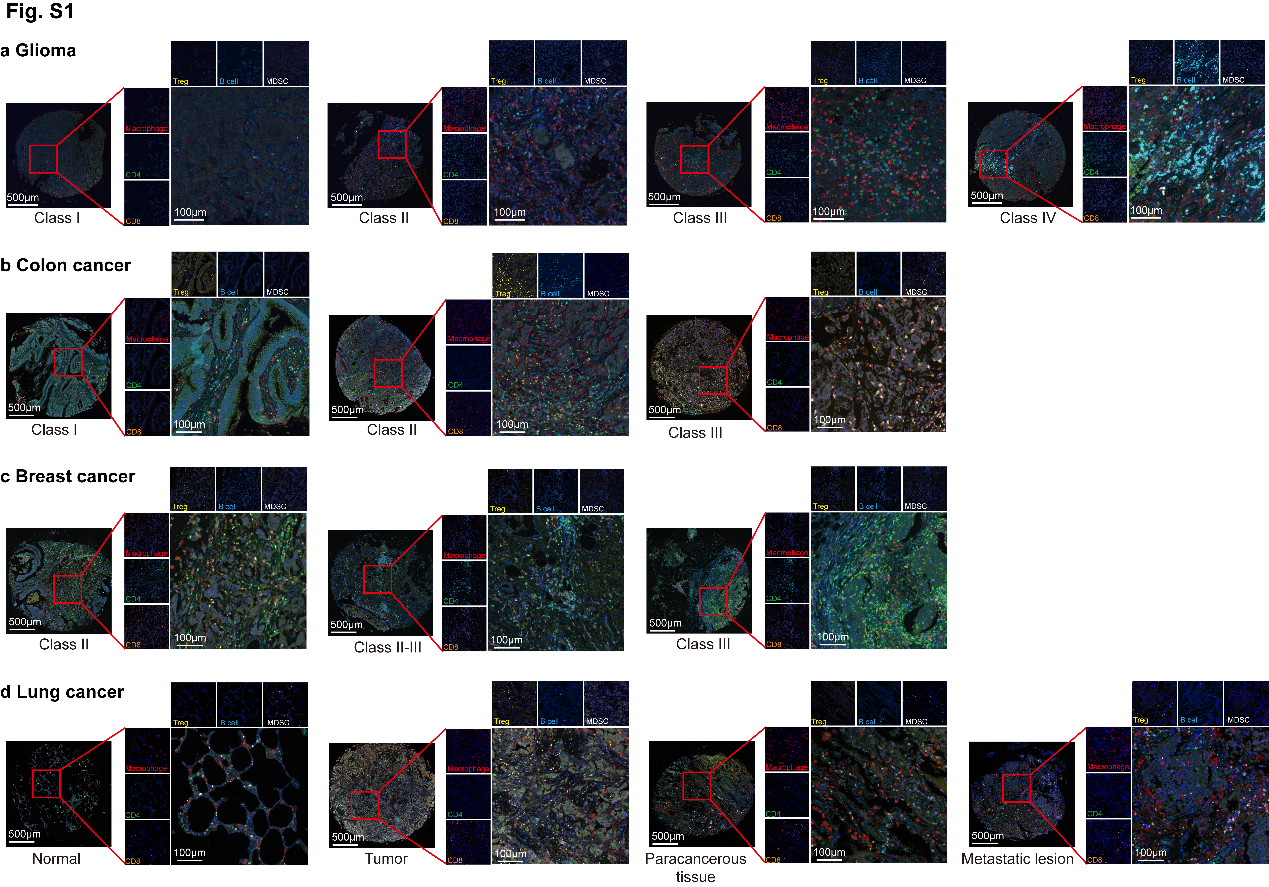


**Fig. S1. The representative images of immune subsets in glioma, colon cancer, breast cancer and lung cancer microarrays by mIHC.** B cells (anti-CD20), CD4^+^ T cells (anti-CD4), CD8^+^ T cells (anti-CD8), Tregs (anti-Foxp3), Macrophage (anti-CD68) and MDSCs (anti-Arginase-1) were identified in tumor tissues.


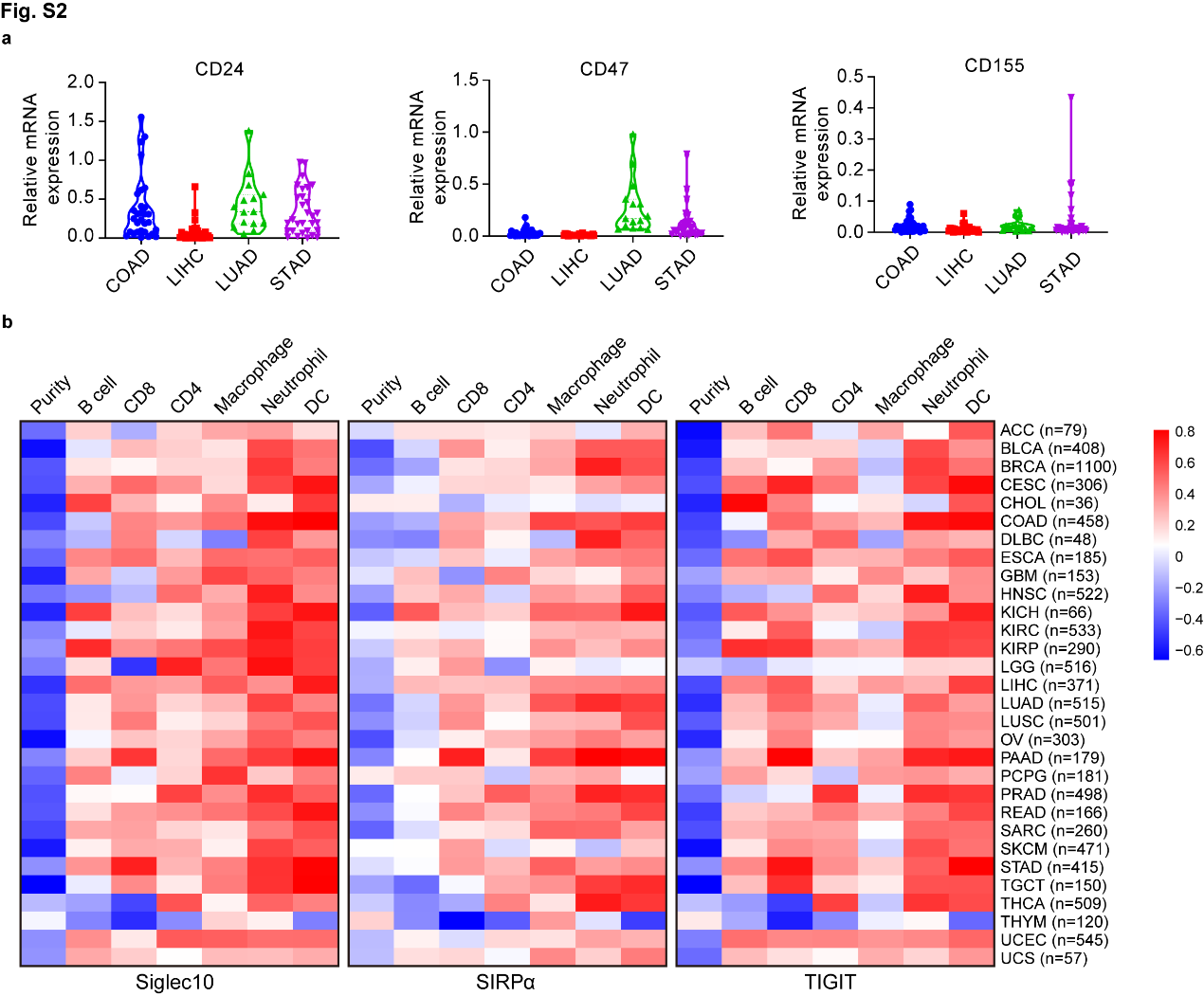


**Fig. S2. The expression of immune checkpoints in the tumor microenvironment. a** The relative mRNA expression of CD24, CD47 and CD155 in COAD, LIHC, LUAD and STAD patient samples in cDNA microarray. **b** Heatmap representing the correlations between checkpoint molecules and infiltrating immune cells, including B cells, CD8^+^ T cells, CD4^+^ T cells, macrophages, neutrophil and dendritic cell. The correlations were determined by TIMER (http://timer.cistrome.org/), which analyzed only the samples from the TCGA database.


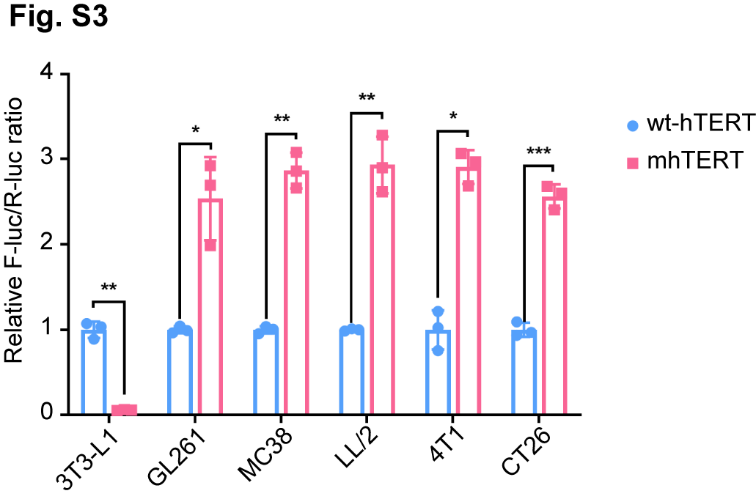


**Fig. S3. Tumor specificity of the m-hTERT promoter.** Tumor-selective activity of the wild-type hTERT promoter (wt-hTERT) and mhTERT promoter was validated using a 48h dual firefly (F-luc) and *Renilla* (R-luc) luciferase reporter gene assay. “F-luc/R-luc”: ratio of firefly/*Renilla* luciferase activities. Data are represented as mean ± SD. (**p*<0.05, ***p*<0.01)


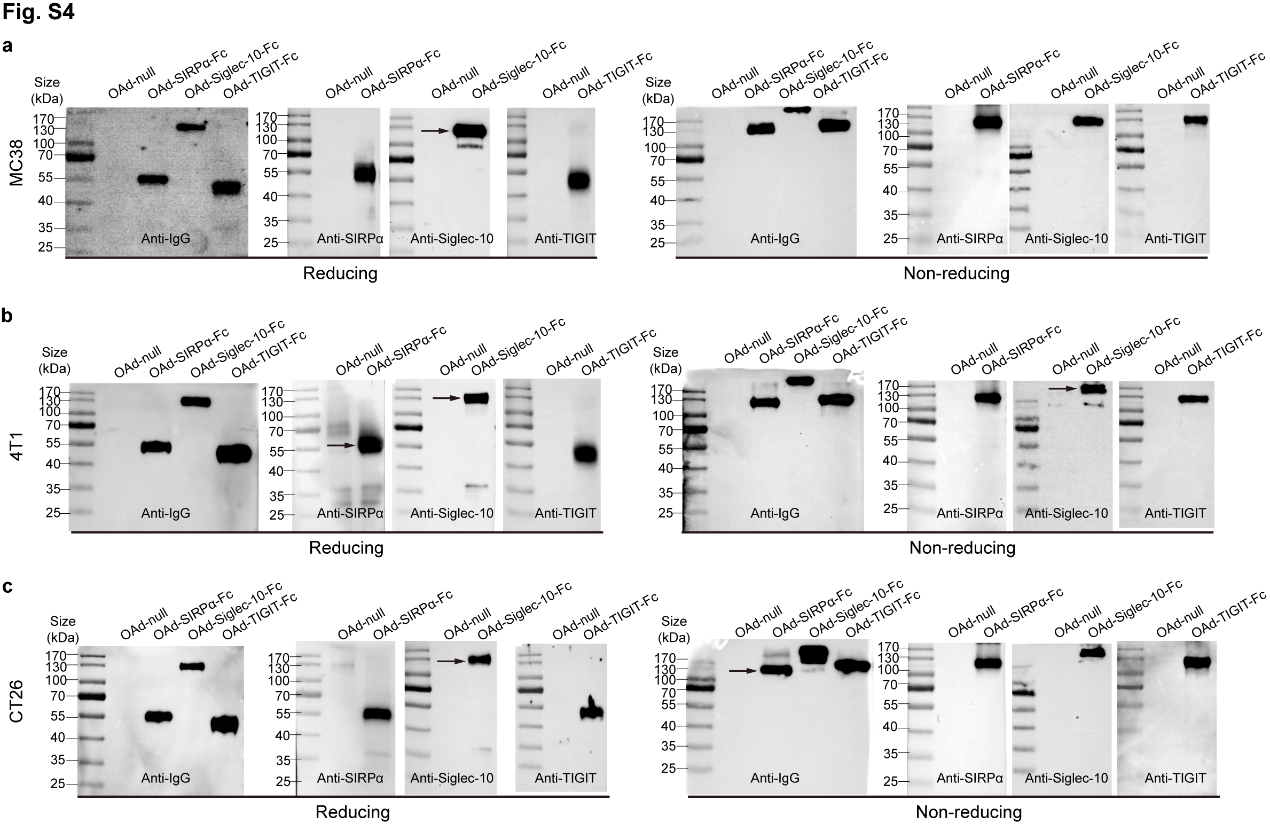


**Fig. S4. Efficient secretion of SIRPα-Fc, Siglec10-Fc and TIGIT-Fc from OAds-infected tumor cells.** The expression and secretion of SIRPα-Fc, Siglec10-Fc and TIGIT-Fc into the supernatant from the indicated OAds-infected MC38 (**a**), 4T1(**b**), and CT26(**c**) tumor cells were detected by western blotting under reducing or nonreducing conditions. These images indicated the full uncut western blots.


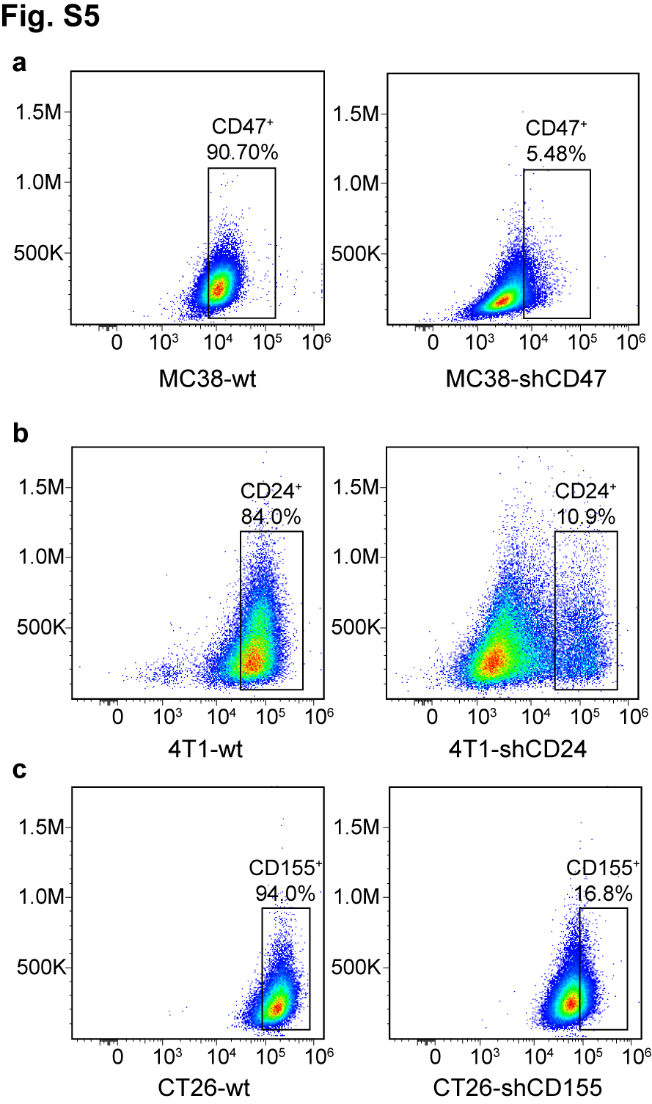


**Fig. S5. The construction of knockdown cell lines using shRNA**. Wild-type MC38, 4T1 and CT26 tumor cells were respectively transduced with CD47-shRNA, CD24-shRNA and CD155-shRNA. These three wild-type tumor cells were all transduced with scramble shRNA as control. The knockdown of CD47 (MC38-shCD47), CD24 (4T1-shCD24) and CD155 (CT26-shCD155) cells were confirmed by flow cytometry.


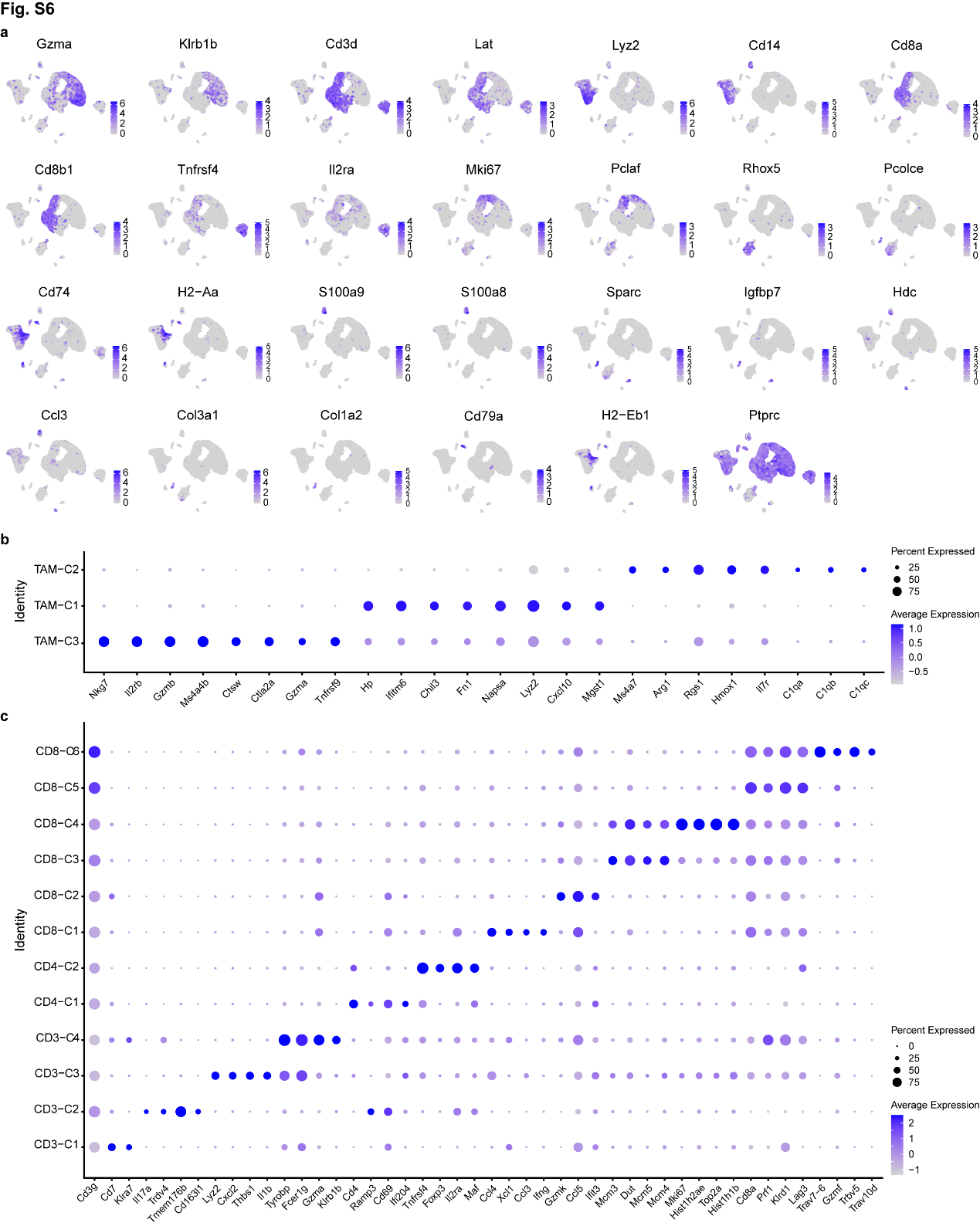


**Fig. S6. Cell type marker in MC38 tumor. a** UMAPs show expression mapping of all 7 cell clusters in MC38 model, all immune cells by *Ptprc*; Non-immune cell by *Rhox5, Pcolce, Sparc, Igfbp7, Col3a1* and *Col1a2*; B cell by *CD79a* and *H2-Eb1*; DC by *CD74* and *H2-Aa*; T cell by *CD3d, Lat, CD8a, CD8b1, Tnfrsf4* and *Il2ra*; macrophage by *Lyz2* and *CD14*; neutrophil by *S100a9, S100a8, Hdc* and *Ccl3*; NK cell by *Gzma* and *Klrb1b*; **b** Macrophage subclusters-associated markers; **c** T cell subclusters-associated markers.


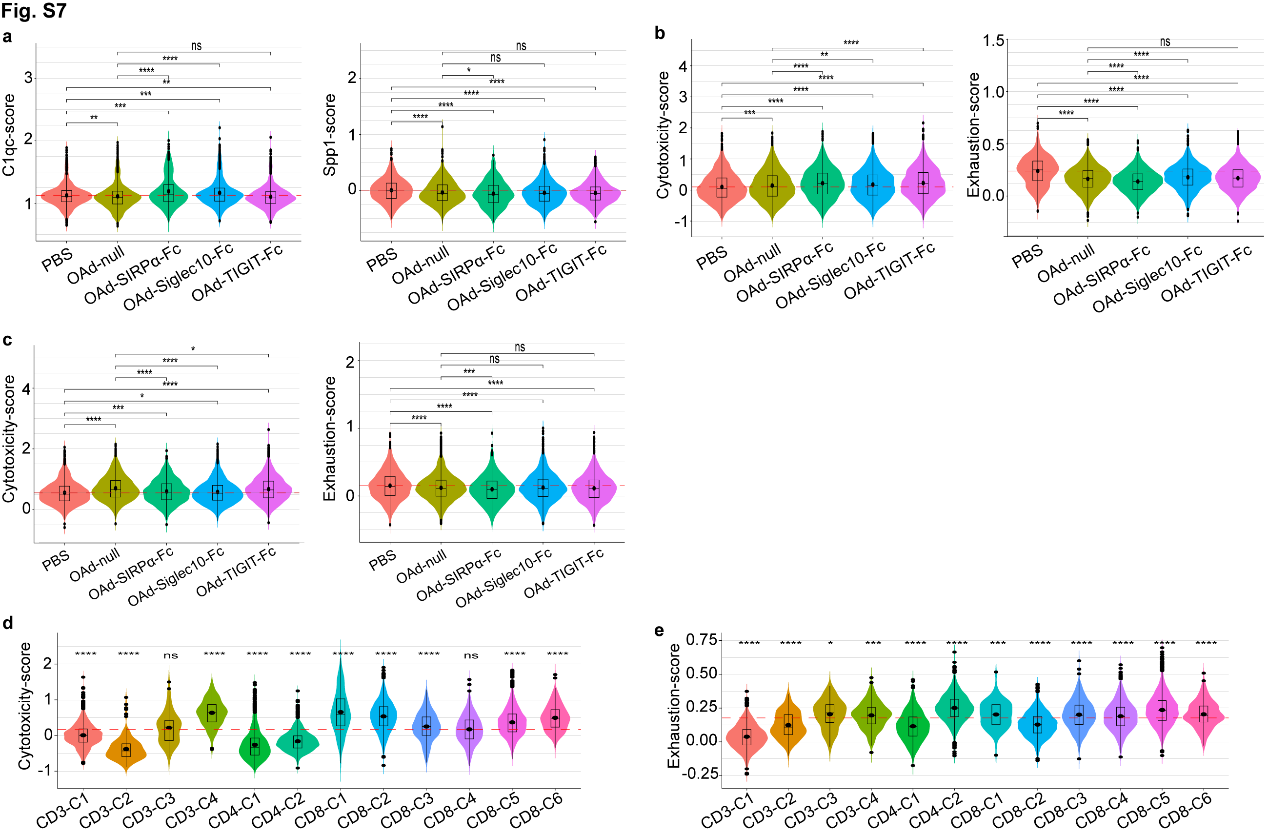


**Fig. S7. RNA expressions of gene signatures associated with therapeutic response to OAds.** C1qc^+^ TAMs and Spp1^+^ TAMs gene signature score (**a**), cytotoxicity and exhausted T cell gene signature score (**b**) and cytotoxicity and exhausted NK cell gene signature score (**c**) of all identified cells in MC38 model. Cytotoxicity (**d**) and exhausted (**e**) T cell gene signature score of T cell subclusters in MC38 model. (ns *p*>0.05, **p*<0.05, ***p*<0.01, ****p*<0.001, *****p*<0.0001)


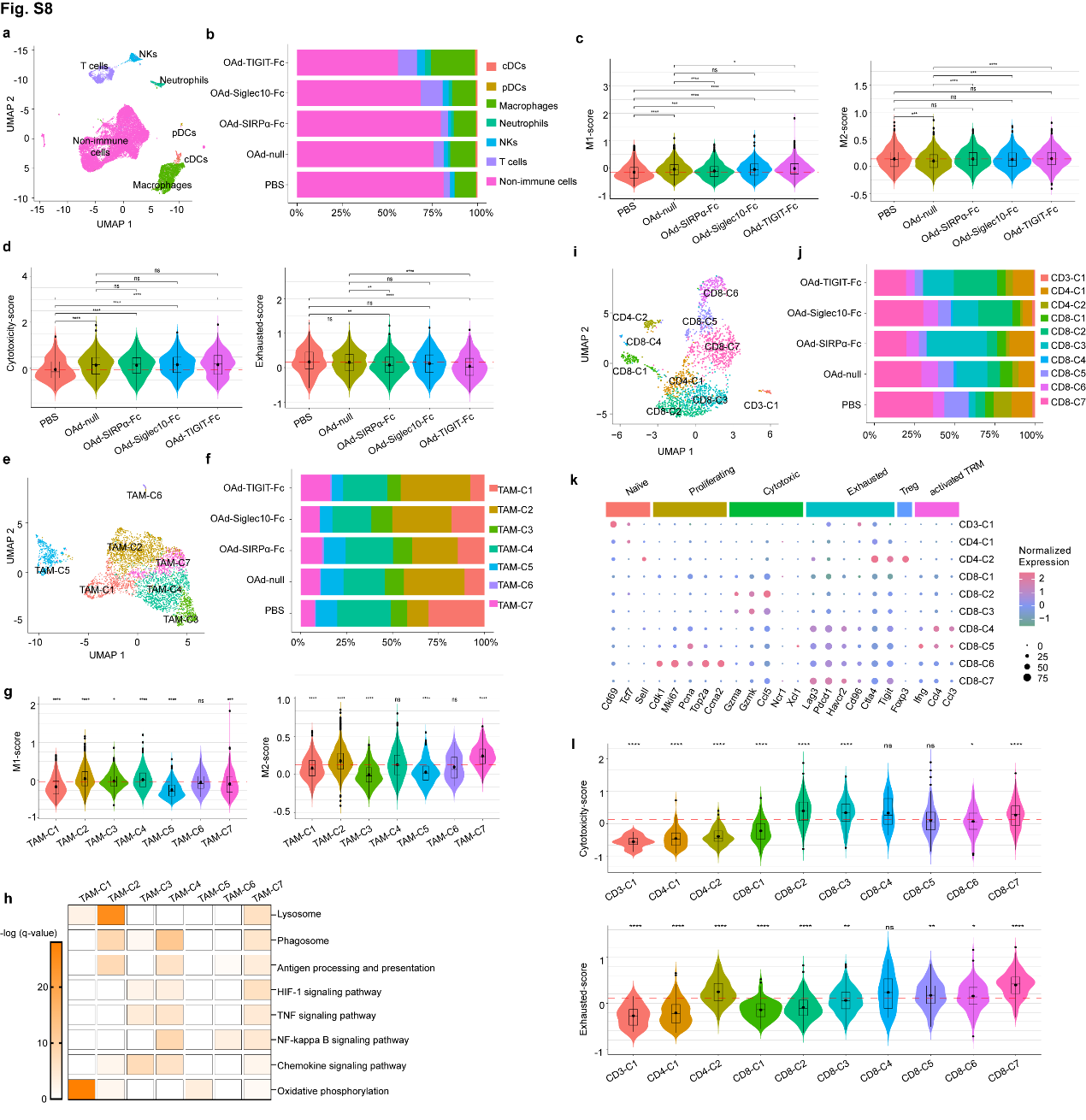


**Fig. S8. Assessment of the functional states of tumor-infiltrating TAMs and T cells in the CT26 model.** CT26 tumor-bearing mice were treated with PBS, OAd, OAd-SIRPα-Fc, OAd-Siglec10-Fc and OAd-TIGIT-Fc. Two days after the third OAds dose, tumor issues were profiled by scRNA-seq. UMAP plot of all single cells in the CT26 model (**a**)and histogram indicating the proportions of cell clusters in tumor tissues (**b**). **c** M1^+^ TAMs and M2^+^ TAMs gene signature score of all identified cell in the CT26 model. **d** Cytotoxicity and exhausted T cell gene signature score of all identified cell in the CT26 model. **e, f** UMAP plots of identified macrophages and frequencies of macrophage subsets from the scRNA-seq analysis. **g** Violin plots showing comparison of M1^+^ score as well as M2^+^ score levels of TAM subclusters. **h** Heatmap of the enriched KEGG pathways in TAMs subclusters. **i, j** UMAP plots of identified T cells and frequencies of T cell subsets from scRNAseq analysis. **k** The relative average expression of canonical markers genes across different T cell clusters. **l** Cytotoxicity and exhausted T cell gene signature score of T cell subclusters. (ns *p*>0.05, **p*<0.05, ***p*<0.01, ****p*<0.001, *****p*<0.0001)


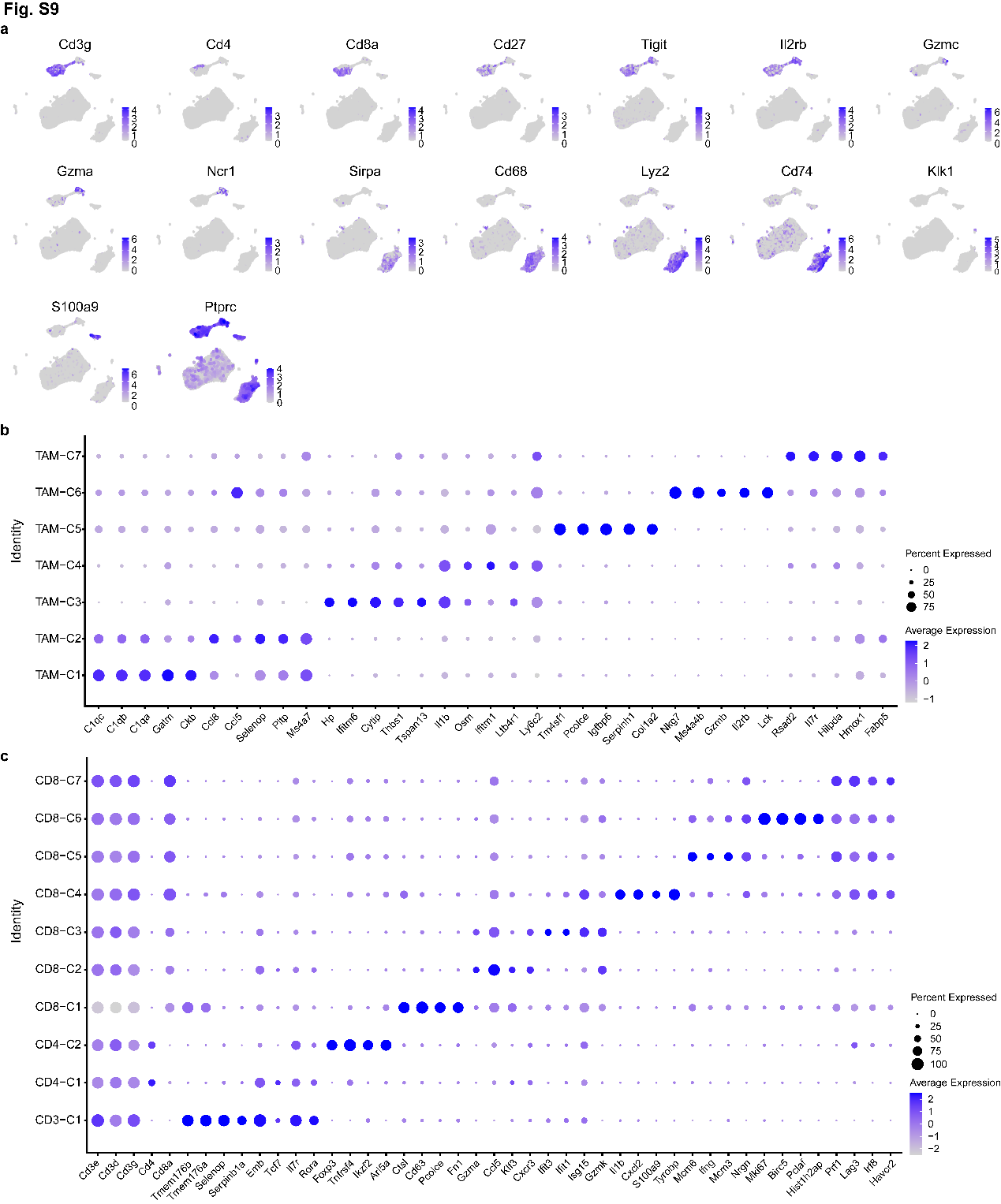


**Fig. S9. Cell type marker in CT26 tumor. a** UMAPs show expression mapping of all 7 cell clusters in CT26 model, all immune cells by *Ptprc*; cDC by *H2-Aa*, *H2-Eb1*, *H2-Ab1*, *CD74* and *H2-DMb2*; pDC by *Klk1*, *Siglech*, *Cox6a2*, *Rnase6* and *Irf8*; macrophage by *Lyz2*, *Apoe*, *Fcer1g*, *CD68* and *Msr1*; neutrophil by *Cxcl2*, *Il1b*, *CCrl2*, *Srgn* and *Ccl3*; NK cell by *Gzma*, *Il2rb*, *prf1*, *Nkg7* and *Ncr1*; T cell by *CD3e*, *CD3g*, *Ms4a4b*, *CD3d* and *Gzmb*. **b** Macrophage subclusters-associated markers. **c** T cell subclusters-associated markers.


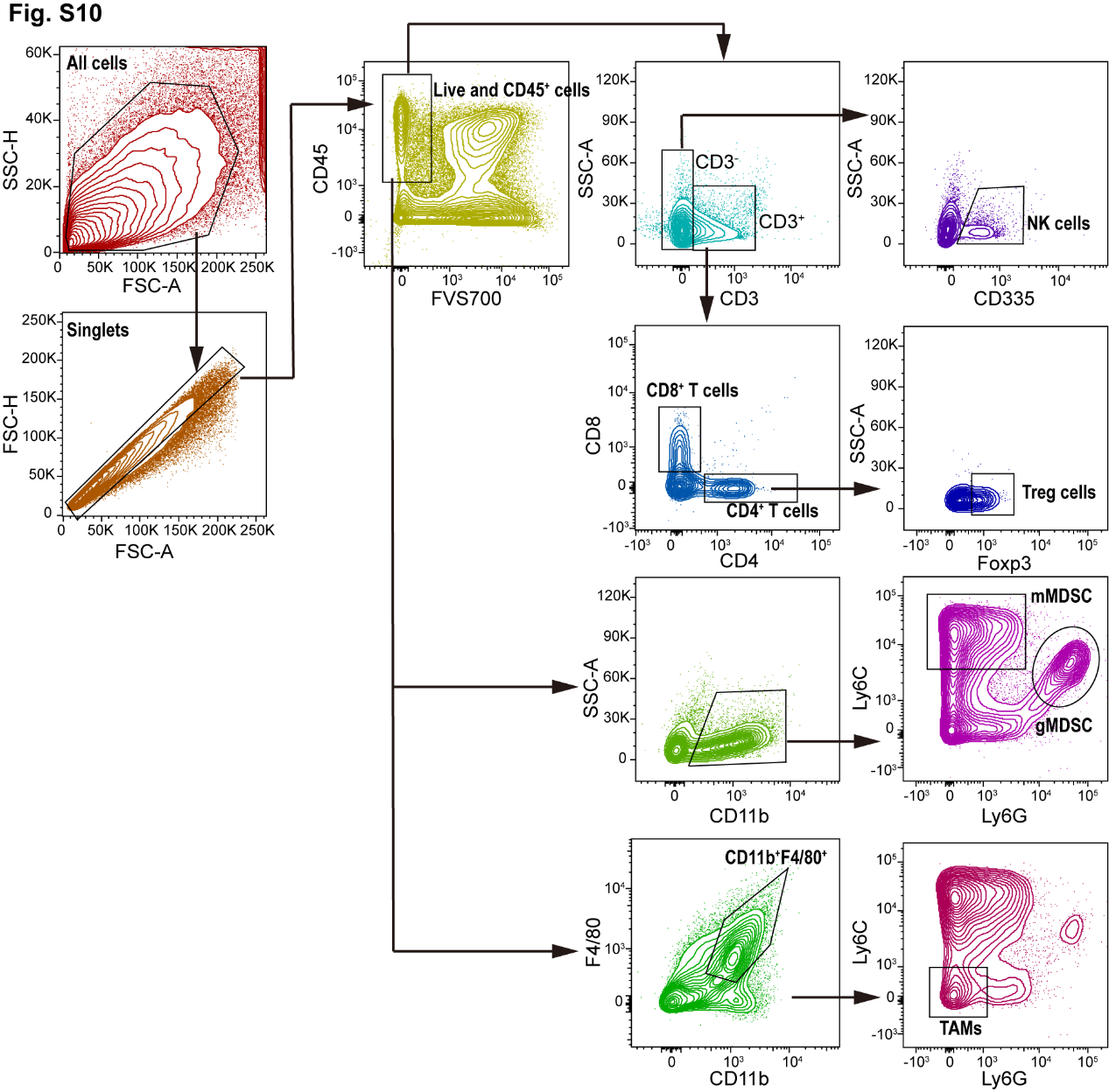


**Fig. S10. Gating strategy for tumor-infiltrating lymphocyte.**


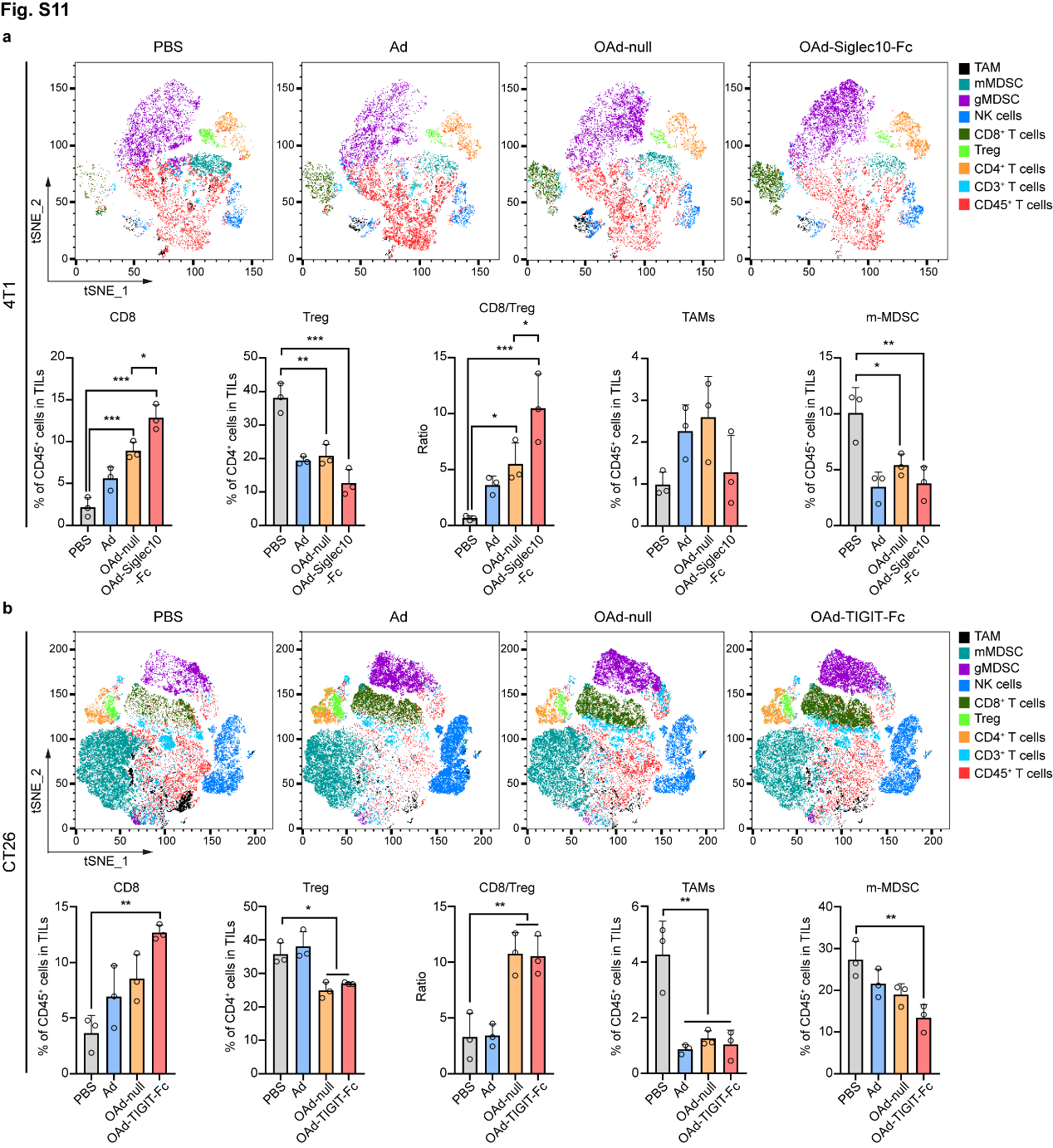


**Fig. S11. Infiltrating Immune cells detected by flow cytometry in 4T1 and CT26 tumors**. Two days after the third injection, the treated tumors were collected and analyzed by flow cytometry to calculate the percentages of infiltrating CD8^+^ T cells, Tregs, TAMs and mMDSCs in 4T1 tumors (**a**) and CT26 tumors (**b**). *n* = 3 mice. Data are represented as mean ± SD. (**p*<0.05, ***p*<0.01, ****p*<0.001)


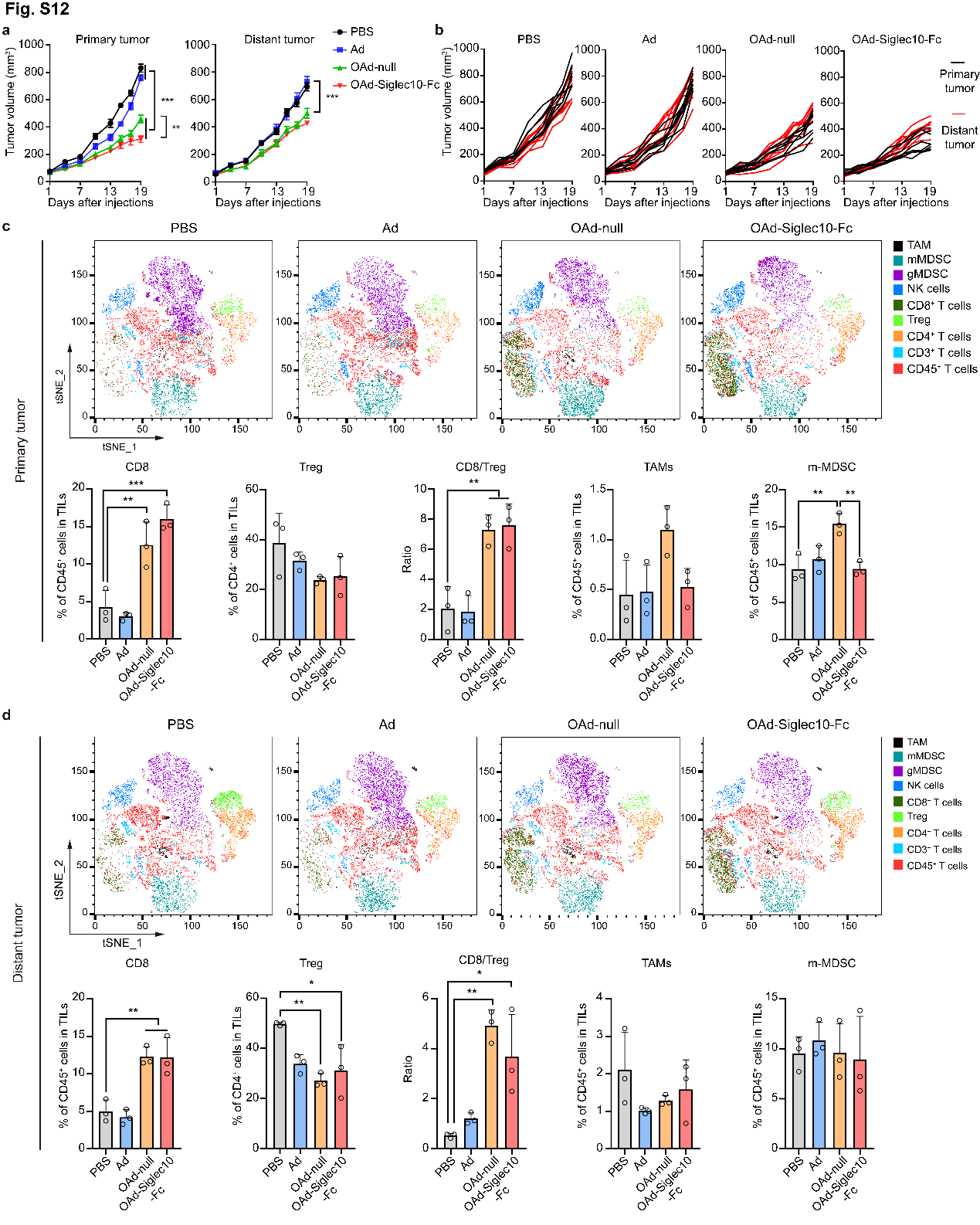


**Fig. S12. Enhanced antitumor activities against untreated distant 4T1 tumors**. **a** Mice were subcutaneously inoculated 4T1 tumor cells in both flanks. After establishment of tumors, the right tumors were intratumorally injected with PBS or Ad, OAd-null, OAd-Siglec10-Fc (3×10^8^ pfu per tumor) at days 1, 4, 7, 10 and 13, respectively. The growth of injected tumors and distant tumors in bilateral 4T1 tumor model. *n* = 8 mice, Data are represented as mean ± SEM. Tumor-infiltrating lymphocyte in injected tumors (**c**) and distant tumors (**d**) were analyzed by flow cytometry. *n* = 3 mice. Data are represented as mean ± SD. (**p*<0.05, ***p*<0.01, ****p*<0.001)


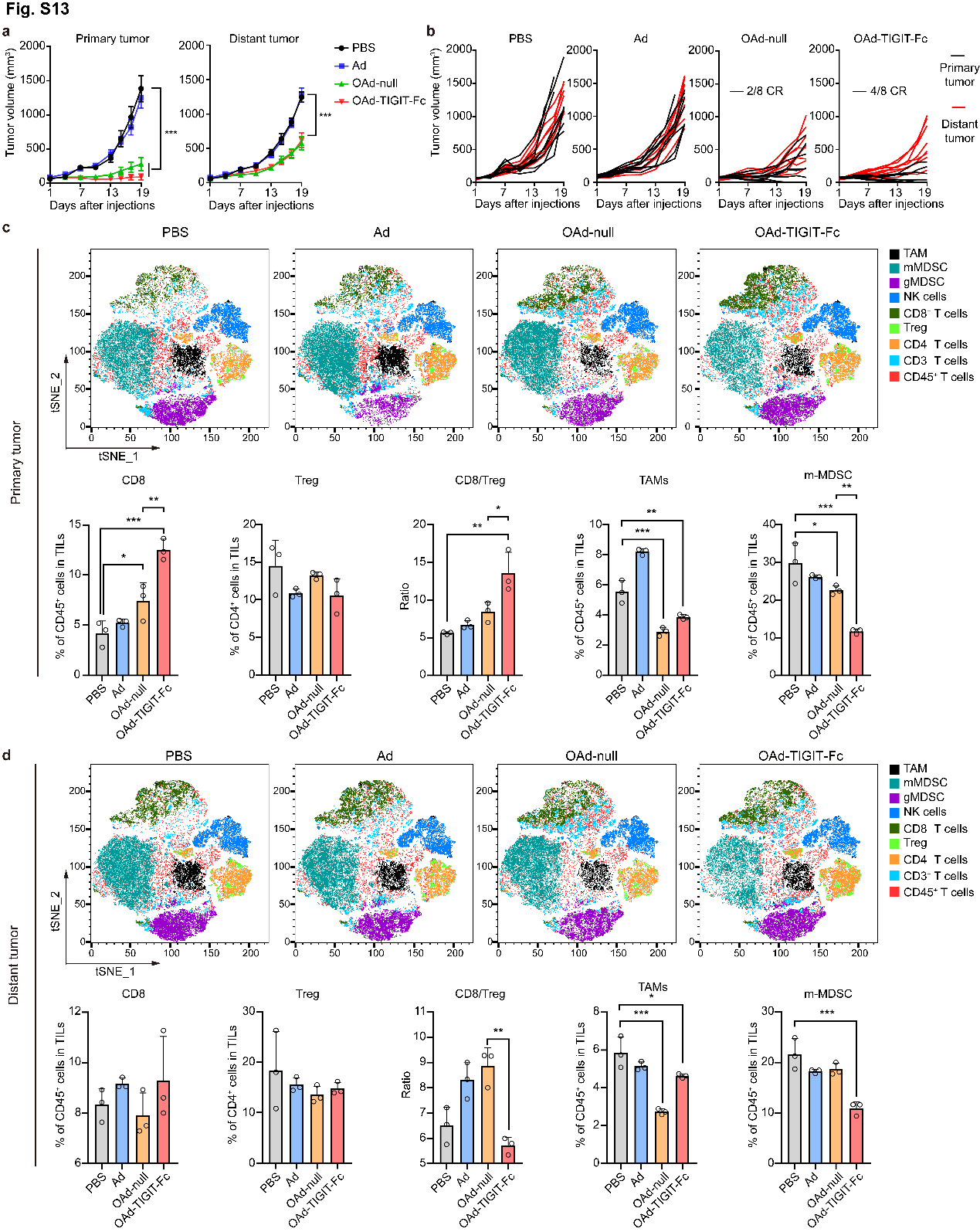


**Fig. S13. Enhanced antitumor activities against untreated distant CT26 tumors**. **a** Mice were subcutaneously inoculated CT26 tumor cells in both flanks. After establishment of tumors, the right tumors were intratumorally injected with PBS or Ad, OAd-null, OAd-TIGIT-Fc (1×10^8^ pfu per tumor) at days 1, 4, 7, 10 and 13, respectively. The growth of injected tumors and distant tumors in bilateral CT26 tumor model. *n* = 8 mice, Data are represented as mean ± SEM. **c, d** TILs in injected tumors (c) and distant tumors (d) were analyzed by flow cytometry. *n* = 3 mice. Data are represented as mean ± SD. (**p*<0.05, ***p*<0.01, ****p*<0.001)


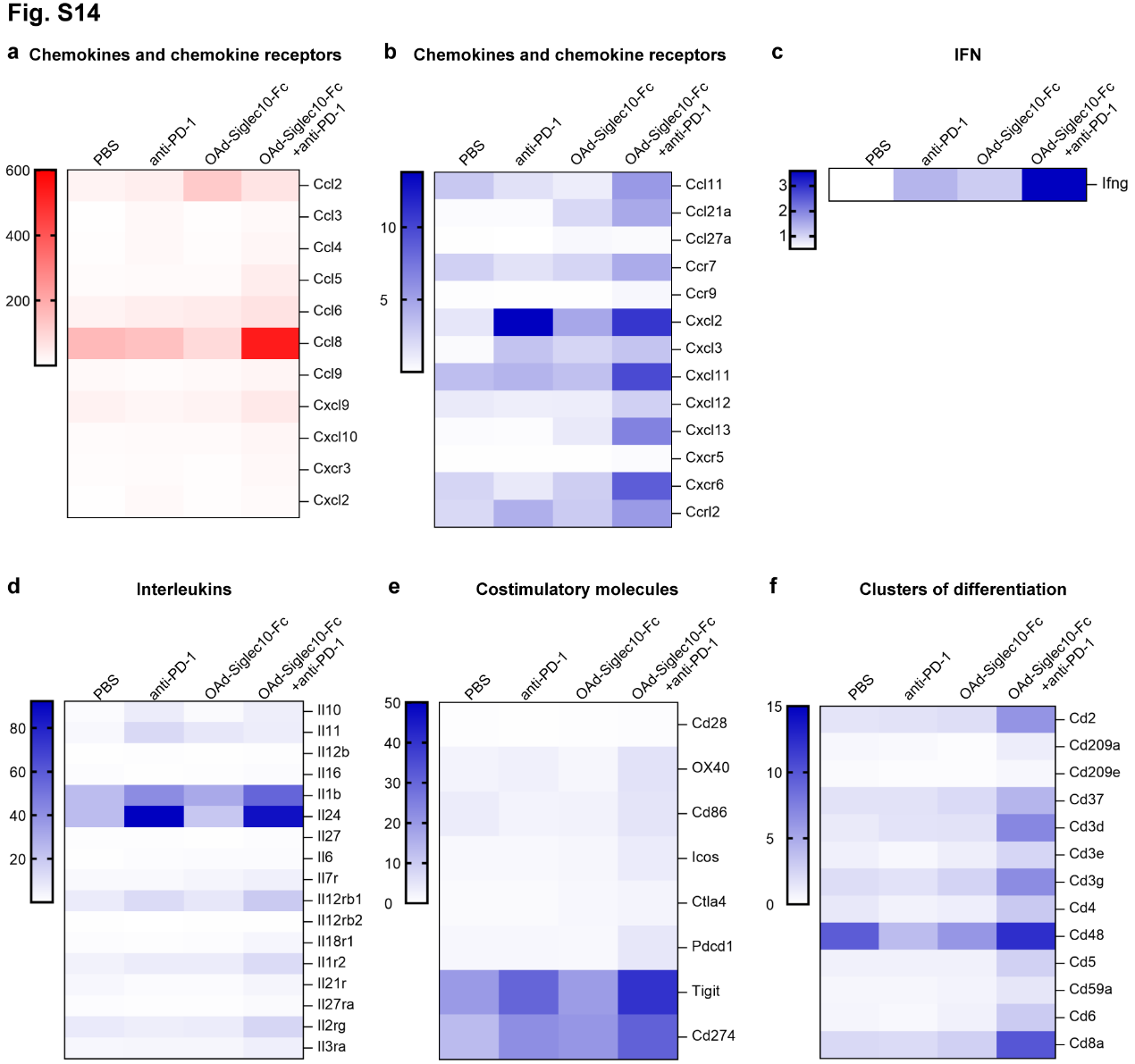


**Fig. S14. Selected a few important up-regulated genes altered by the combination therapy.** Heatmaps showing up-regulated genes of chemokines and chemokine receptors, cytokines, Ifng, interleukins, costimulatory molecules, and clusters of differentiation altered by the combination therapy.

Table S1.

Detailed information of tumor and healthy issues for TCGA datasets.

| **TCGA Study Abbreviation** | **TCGA Study Name** | **Patient tumors analyzed (n)** | **Healthy samples analyzed (n)** |
| --- | --- | --- | --- |
| ACC | Adrenocortical carcinoma | 77 | 128 |
| BLCA | Bladder Urothelial Carcinoma | 404 | 28 |
| BRCA | Breast invasive carcinoma | 1085 | 291 |
| CESC | Cervical squamous cell carcinoma and endocervical adenocarcinoma | 306 | 13 |
| CHOL | Cholangio carcinoma | 36 | 9 |
| COAD | Colon adenocarcinoma | 275 | 349 |
| DLBC | Lymphoid Neoplasm Diffuse Large B-cell Lymphoma | 47 | 337 |
| ESCA | Esophageal carcinoma | 182 | 286 |
| GBM | Glioblastoma multiforme | 163 | 207 |
| HNSC | Head and Neck squamous cell carcinoma | 519 | 44 |
| KICH | Kidney Chromophobe | 66 | 53 |
| KIRC | Kidney renal clear cell carcinoma | 523 | 100 |
| KIRP | Kidney renal papillary cell carcinoma | 286 | 60 |
| LAML | Acute Myeloid Leukemia | 173 | 70 |
| LGG | Brain Lower Grade Glioma | 518 | 207 |
| LIHC | Liver hepatocellular carcinoma | 369 | 160 |
| LUAD | Lung adenocarcinoma | 483 | 347 |
| LUSC | Lung squamous cell carcinoma | 486 | 338 |
| OV | Ovarian serous cystadenocarcinoma | 426 | 88 |
| PAAD | Pancreatic adenocarcinoma | 179 | 171 |
| PCPG | Pheochromocytoma and Paraganglioma | 182 | 3 |
| PRAD | Prostate adenocarcinoma | 492 | 152 |
| READ | Rectum adenocarcinoma | 92 | 318 |
| SARC | Sarcoma | 262 | 2 |
| SKCM | Skin Cutaneous Melanoma | 461 | 558 |
| STAD | Stomach adenocarcinoma | 407 | 211 |
| TGCT | Testicular Germ Cell Tumors | 137 | 165 |
| THCA | Thyroid carcinoma | 512 | 337 |
| THYM | Thymoma | 118 | 339 |
| UCEC | Uterine Corpus Endometrial Carcinoma | 174 | 91 |
| UCS | Uterine Carcinosarcoma | 57 | 78 |

Table S2.

Gene signatures for scoring.

| **Types** | **Gene signatures** |
| --- | --- |
| M1 score | *Ccl5, Ccr7, Cd40, Cd86, Cxcl9, Cxcl10, Ccl8, Ido1, Il1a, Il1b, Il6, Irf1, Irf5, Kynu* |
| M2 score | *Ccl4, Ccl13, Ccl18, Ccl20, Ccl22, Cd276, Clec7a, Ctsa, Ctsb, Ctsd, Fn1, Irf4, Lyve1, Mmp9, Mmp14, Mmp19, Tgfb1, Tgfb2, Tgfb3, Tnfsf8, Tnfsf12, Vegfa, Vegfb, Vegfc^1,2^* |
| C1qc^+^ score | *C1qa, C1qb, C1qc, Itm2b, Ms4a6a, Ctsc, Tbxas1, Tmem176b, Syngr2, Arhgdib, Tmem176a, Ucp2, Capzb, Maf, Trem2, Msr1* |
| Spp1^+^ score | *Spp1, Pcsk5, Slc11a1, Vcan, Slc25a37, Flna, Upp1, Bcl6, Aqp9, Timp1, Vegfa, Adm, Marco, Fn1, Il1rn^1^* |
| Cytotoxicity score forT cell and NK cell clusters | *Prf1, Ifng, Ccl4, Gzmk, Gzma, Gzmb, Cd44, Ccl5, Dusp2, Klrb1, Klrd1, Ctsw, H2-Aa* |
| Exhausted score for T cell and NK cell clusters | *Cxcl13, Havcr2, Pdcd1, Tigit, Lag3, Ctla4, Layn, Rbpj, Vcam1, Gzmb, Tox, Myo7a*^3-5^ |
| naïve score for T cells and NK cell clusters | *Ccr7, Lef1, Tcf7,* *Sell*^6^ |

Table S3.

Plasmid DNA mixed for transfection.

|  | **pRL-TK**  **(ng)** | **pGL3-basic**  **(ng)** | **pGL3-wt-hTERT**  **(ng)** | **pGL3-m-hTERT**  **(ng)** |
| --- | --- | --- | --- | --- |
| Control | 0 | 0 | 0 | 0 |
| Basic | 10 | 100 | 0 | 0 |
| wt-hTERT | 10 | 0 | 100 | 0 |
| m-hTERT | 10 | 0 | 0 | 100 |

Table S4.

shRNA targeting sequence against mouse CD47, CD24, CD155.

| **Target** | **sequence** |
| --- | --- |
| CD47 shRNA | Sense:5’-CCGGCACCGAAGAAATGTTTGTGAACTCGAGTTCACAAACATTTCTTCGGTGTTTTT-3’ |
|  | Antisense:3’- aattcAAAAACACCGAAGAAATGTTTGTGAACTCGAGTTCACAAACATTTCTTCGGTG-5’ |
| CD24 shRNA | Sense:5’-CCGGTGTTGCACCGTTTCCCGGTAACTCGAGTTACCGGGAAACGGTGCAACATTTTT-3’ |
|  | Antisense:3’-aattcAAAAATGTTGCACCGTTTCCCGGTAACTCGAGTTACCGGGAAACGGTGCAACA-5’ |
| CD155 shRNA | Sense:5’-CCGGCGTCCAGTATTCATCTGTGAACTCGAGTTCACAGATGAATACTGGACGTTTTT-3’ |
|  | Antisense:3’-aattcAAAAACGTCCAGTATTCATCTGTGAACTCGAGTTCACAGATGAATACTGGACG-5’ |

Table S5.

Cell line details

| **Cell line** | **Supplier** | **Strain** | **Sex** | **Tissur of origin** | **Culture conditions** | **Inoculum** |
| --- | --- | --- | --- | --- | --- | --- |
| MC38 | Kerafast | C57BL/6J | Female | Colorectal | DMEM, 10%FBS | 1e^6^ |
| 4T1 | ATCC | Balb/c | Female | Colon | RPMI 1640, 10%FBS | 1e^6^ |
| CT26 | ATCC | Balb/c | Female | Breast | RPMI 1640, 10%FBS | 1.5e^6^ |

Table S6.

Information for the Validation of Primary Antibodies

| Antibody | Anti-CD20 | Anti-CD4 | Anti-FoxP3 | Anti-CD8 | Anti-CD68 | Anti-Arginase-1 | / |
| --- | --- | --- | --- | --- | --- | --- | --- |
| Clone | E7B7T | EP204 | D2W8E | C8/144B | D4B9C | D4E3M™ | / |
| Vendor | CST | CST | CST | CST | CST | CST | / |
| Catalog | 48750 | 48274 | 98377 | 70306 | 76437 | 93668 | / |
| Antibody dilution | 1:400 | 1:200 | 1:100 | 1:500 | 1:800 | 1:200 | / |
| TSA-Dye | Opal 480 | Opal 520 | Opal 570 | Opal 620 | Opal 690 | Opal 780 | DAPI |
| Order | 1 | 2 | 3 | 4 | 5 | 6 | 7 |

References

1 Zhang, L. *et al.* Single-Cell Analyses Inform Mechanisms of Myeloid-Targeted Therapies in Colon Cancer. *Cell*. **181**, 442-459 e429, (2020).

2 Azizi, E. *et al.* Single-Cell Map of Diverse Immune Phenotypes in the Breast Tumor Microenvironment. *Cell*. **174**, 1293-1308 e1236, (2018).

3 Zhang, L. *et al.* Lineage tracking reveals dynamic relationships of T cells in colorectal cancer. *Nature*. **564**, 268-272, (2018).

4 Zheng, C. *et al.* Landscape of Infiltrating T Cells in Liver Cancer Revealed by Single-Cell Sequencing. *Cell*. **169**, 1342-1356 e1316, (2017).

5 Zhang, Y. *et al.* Single-cell analyses reveal key immune cell subsets associated with response to PD-L1 blockade in triple-negative breast cancer. *Cancer Cell*. **39**, 1578-1593 e1578, (2021).

6 Guo, X. *et al.* Publisher Correction: Global characterization of T cells in non-small-cell lung cancer by single-cell sequencing. *Nat Med*. **24**, 1628, (2018).
